# Immunomodulatory effects of modified Liu-Wei-Di-Huang-Wan Traditional Chinese medicine on allergic asthmatic mice

**DOI:** 10.1186/s13223-023-00792-5

**Published:** 2023-04-26

**Authors:** Jaw-Ji Tsai, Chung-Yang Yen, Chun-Hsien Hsu, Sheng-Jie Yu, Chao-Hsien Chen, En-Chih Liao

**Affiliations:** 1 Division of Allergy, Immunology and Rheumatology, Department of Internal Medicine, Asia University Hospital, Taichung, Taiwan; 2 Department of Dermatology, Taichung Veterans General Hospital, Taichung, Taiwan; 3 School of Medicine, National Yang-Ming University, Taipei, Taiwan; 4 School of Medicine, College of Medicine, Fu Jen Catholic University, New Taipei, Taiwan; 5 Department of Family Medicine, Heping Fuyou Branch, Taipei City Hospital, Taipei, Taiwan; 6 Department of Family Medicine, Cardinal Tien Hospital, New Taipei, Taiwan; 7 Department of Medical Education and Research, Taichung Veterans General Hospital, Taichung, Taiwan; 8 Division of Pulmonary and Critical Care Medicine, Department of Internal Medicine, MacKay Memorial Hospital, Taipei, Taiwan; 9 Department of Medicine, MacKay Medical College, No.46, Sec. 3, Zhongzheng Rd., Sanzhi Dist., New Taipei City, 252 Taiwan; 10 Institute of Biomedical Sciences, MacKay Medical College, New Taipei City, Taiwan

**Keywords:** Allergic asthma, House dust mite, *Dermatophagoides pteronyssinus*, Der p 2, Liu-Wei-Di-Huang-Wan (LWDHW), Immunotherapy

## Abstract

**Background:**

Allergic asthma occurs worldwide and is particularly prevalent in westernized countries characterized by chronic airway inflammation resulting in airway hyperresponsiveness. The house dust mites (HDM) including *Dermatophagoides pteronyssinus* are major sources of sensitization and triggering allergic symptoms in asthmatic patients. The Der p 2 is a major allergen and the predominant source of causative respiratory disorders which induce airway inflammation and bronchial constriction in mite-allergic patients. Few studies evaluate the ameliorating effects of modified Liu-Wei-Di-Huang-Wan (modified LWDHW) on allergic asthma.

**Methods:**

This study aimed to investigate the immunological mechanisms of modified LWDHW on the reductions of airway inflammation, signal transduction, inflammatory cytokine production, Th2 cell proliferation, and bronchial obstruction in Der p 2-induced asthmatic mice.

**Results:**

At least ten active ingredients were contained in the formula of modified LWDHW- 1217A and 1217B. Results showed that the immunoglobulin generations (Der p 2 specific- IgE and IgG1), inflammatory cytokine productions (IL-5 and IL-13) in the Sera and BALF could be down-regulated, and the Th1-cytokine productions (IL-12 and IFN-γ) be increased after immunotherapy with modified LWDHW of 1217A or 1217B. The inflammatory cell infiltrations (macrophages, eosinophils, and neutrophils) in the airway and the expressions of T_H_2-related genes (*IL-4*, *IL-5*, and *IL-13*), T_H_2-related transcription factor (*GATA-3*), and neutrophil chemotactic chemokine (*IL-8*) in the lung tissue of asthmatic mice were significantly decreased after the immunotherapy. The Th1/Th2 polarization had been identified that the IL-4^+^/CD4^+^ T cells were downregulated and IFN-γ^+^/CD4^+^ T cells were increased. The airway hyperresponsiveness to methacholine inhalation of Penh values was significantly decreased in the treated groups. There were significant improvements in the bronchus histopathology after immunotherapy with 1217A or 1217B which were evaluated by tracheal thickness, inflammatory cell count, and tracheal rupture of mouse lung.

**Conclusion:**

It revealed that 1217A or 1217B could regulate the immune responses and improve pulmonary function. Data suggests that modified LWDHW of 1217A or 1217B have the potential for use as a therapeutic intervention for the treatment of mite allergen Der p 2-induced allergic asthma.

## Background

More and more evidence suggests that the occurrence of allergic diseases is a serious public health concern, and the allergy prevalence increased widespread [1]. Asthma is one of the most common allergic diseases worldwide and there is accumulating evidence indicating that its prevalence is increasing, and pathogenesis is associated with allergen exposure [2]. Although the reasons for this increased incidence are fully clarified, there is a clear change in patients’ physical state associated with abnormal immunological reactions which manifest as chronic inflammation and tracheal hypersensitivity [3]. These allergic symptoms or chronic disorders of asthma most commonly be disposed of preventing disease progression are relying on medicine administration such as steroid-related drugs [4]. Some allergic patients exhibit steroid resistance, so they are not suitable for corticosteroid therapies; therefore, we must seek the alternative method of therapeutic targets and develop more effective therapies for these allergic or chronic asthma patients.

The most important risk factors in asthma are allergies caused by allergens. Among these allergy triggers, the inhalant allergens of mites are the most common and considerable triggers of allergic diseases in genetically predisposed patients [5]. There are numerous aeroallergens present in residential circumstances and the most common aeroallergen is the house dust mite (HDM) [6]. Among the HDM, the most prevalent allergen sources are *Dermatophagoides pteronyssinus* and *D. farinae*, especially *Dermatophagoides pteronyssinus,* which are known as a considerable source for allergen sensitization and a major risk factor for allergic symptoms in asthmatic patients [7]. Inhaled HDM allergens interact with airway epithelial cells and bring about epithelium damage, the following contribute to airway remodeling and inflammatory mediators release [8]. *D. pteronyssinus* occurs at high levels of infestation worldwide and with high IgE frequency, which the mite extracts have indicated over 36 different allergenic components can induce IgE in patients and about 32 allergens been identified [9]. Among these allergens that have been characterized and identified from *D. pteronyssinus,* the group II allergen-Der p 2 has been reported to possess the highest IgE-binding frequency in general and it belongs to non-proteolytic proteins [10]. The allergen Der p 2 has been evidenced to display structural homology and functional similarity with myeloid differentiation factor-2 (MD-2) protein that initiates responsiveness to lipopolysaccharide (LPS) integrate with toll-like receptor (TLR)-4 [11].

Allergen-induced asthma of animal models mimicking the major features of human asthma has been reported in several studies to clarify the pathogenesis mechanisms, including immunoglobulin E (IgE) production, airway hyperresponsiveness to methacholine, airway inflammation, inflammatory cell infiltration in bronchoalveolar lavage (BAL) and lung pathology [12]. The Der p 2-induced allergic airway inflammation has been established in our previous study that the leukocyte subpopulation (neutrophils and eosinophils) in BAL, specific immunoglobulin (IgE), inflammatory cytokine (IL-5), pulmonary function, and lung pathology significantly changed in the Der p 2-sensitized and challenged mice [13]. The HDM-induced asthma animal model has been reported to be useful for the evaluation of the effectiveness of immunotherapy [14]. Intraperitoneal sensitization followed by intratracheal challenge with mite extract can induce airway inflammation, tracheal rupture, and tracheal thickening in the asthmatic mouse model [15]; therefore, this asthmatic model is suitable to evaluate the therapeutic effectiveness of candidate medicine like Traditional Chinese Medicine (TCM) on mite-induced allergic responses [15].

Many effective treatments or therapeutic strategies for asthma have been established recently such as pharmacological treatment (bronchodilator or macrolides), non-pharmacological treatment (bronchial thermoplasty), and novel pharmacological agents (CXCR2 antagonist, anti-TNF-α, or anti-IL-6) [16]. Most of these strategies not only relieve allergic symptoms but also alter the immunological status, resulting in the number of Th2 lymphocytes or responses being decreased in asthmatic patients. Patients diagnosed with severe asthma are the administration of inhaled corticosteroids for a long period and with high consumption of healthcare resources and treatment time [17]. However, steroid administration for long-term usage would cause side effects such as Musculoskeletal, gastrointestinal, cardiovascular, endocrine, neuropsychiatric, dermatologic, ocular, and immunologic side effects are all possible [18]. Thus, a new strategy for allergic diseases with alternative treatment is necessary for avoiding excessive or prolonged usage of corticosteroids.

The stratagem of avoiding the incidence of allergic diseases includes allergen avoidance, pharmacotherapy, and allergen-specific immunotherapy. However, it is not easy for avoiding exposure to mite allergens, because dust mites are prevalent in the residential environment worldwide, especially in areas full of human skin dander such as carpets, mattresses, and pillows. Overuse of corticosteroids is also prone to cause a series of side effects or steroid resistance. Allergen-specific immunotherapy intends to decrease the occurrence of hypersensitive symptoms triggered by relevant allergens. However, there are still many unexpected probabilities such as unpredictable anaphylactic reactions, the effective dose varies among patients, and wasting time and costs during the administration of allergen-specific immunotherapy.

Traditional Chinese Medicine (TCM) seems to be a better therapeutic effect and has been used to treat acute and chronic asthma in Asia, national cohort studies showed that the Emergency Room visits and hospitalization of patients who received a combination of conventional therapy with integrated TCM were lower than those who underwent conventional therapy alone [19]. It is not easy for physicians who were trained in modern Western medicine to trust the therapeutic effects of TCM on allergy confidently without scientific support of research data. However, there is a paucity of evidence-based studies demonstrating mechanisms by which TCM could be useful for asthmatic patients.

Some active compounds had been isolated from TCM which with pharmacological effects on inflammatory diseases, such as anti-inflammatory and immunomodulatory effects [20, 21]. The Yam (*Dioscorea*) has been used as a health food and in Chinese medicine because of its nutritional fortification, anti-tussive, expectorant, and anti-inflammatory effects by inhibiting the NF-κB pathway. Inhibition of NF-κB subsequently down-regulates inflammatory markers such as COX-2, iNOS, TNF-α, and IL-1β, therefore these effects may be attributed to the antioxidant and anti-inflammatory nature of Yam (*Dioscorea*) [22]. The lipopolysaccharide (LPS)-induced lung inflammation of acute lung injury can be attenuated by extracts of *Alismatis Rhizoma* tuber powder, which is associated with differential regulation of NF-κB and nuclear factor- erythroid 2 related factor 2 (Nrf2) [23]. Results suggest *Alismatis Rhizoma* extract is involved in Nrf2 activation and suppression of inflammation so that it represents the anti-inflammatory effect and can be developed as a potential therapeutics for lung inflammation [23]. *Scutellaria baicalensis* (Skullcap) has been widely used as a traditional herbal medicine owing to its anti-inflammatory properties, such as its active compound wogonin can suppress IL-4 production *ex vivo* [24]. The ovalbumin (OVA)-induced Th2 immune responses, especially IgE and IL-5 production can be down-regulated by wogonin from skullcap in vivo, results suggest the wogonin acting as an active component of *S. baicalensis* can be applied as a therapeutic agent for IgE- or IL-5 mediated allergic disorders [24]. Active compounds isolated from *Scutellaria baicalensis* include wogonin and baicalin that inhibit the inflammatory response through inhibition of cyclooxygenase-2 (COX-2) gene expression through blockade of CCAAT/Enhancer Binding Protein Beta (C/EBP-Beta) DNA binding activity [24, 25].

However, there are few studies on the use of an animal model to evaluate the ameliorating effects of modified Liu-Wei-Di-Huang-Wan (modified LWDHW) on allergic asthma. The purpose of this study was to investigate the immunological mechanisms of modified LWDHW on the inhibitory effects of airway inflammation, signal transduction, inflammatory cytokine production, Th2 cell proliferation, and bronchial obstruction. This study aimed to explore the TCM of modified LWDHW whether with therapeutic potential for treating allergic asthma in Der p 2-sensitized mice.

## Materials and methods

### Animals

Male BALB/c mice with ages between 6 and 8 weeks old were purchased from the National Laboratory Breeding Research Center in Taiwan and housed at Taichung Veterans General Hospital (TCVGH), Taichung, Taiwan, in a specific pathogen-free environment. Mice were kept at a constant temperature of 23 ± 1 °C, relative humidity of 55 ± 5%, and with the light/dark transition (light: dark = 12 h: 12 h). Mice were divided into five groups (Naïve, saline, Dexamethasone, 1217A, and 1217B), and each group containing six mice was caged separately according to their experiment planning. The experimental procedures were approved by the Animal Committee of the Taichung Veterans General Hospital (La-99784). The whole animal experiments were performed in three independent replicates to verify the scientific phenomenon, all groups were performed in different cages in the same animal center.

### Experimental schedule

**Fig. 1:**
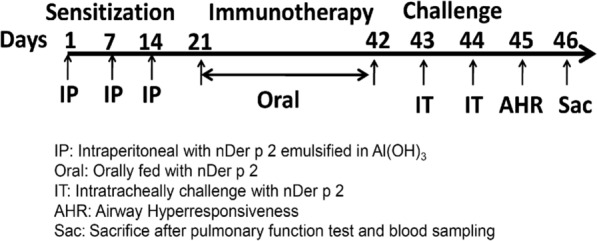
Experimental schedule for intraperitoneal sensitization, immunotherapy, and intratracheal challenge. BALB/c mice were IP-sensitized with 5 µg nDer p 2 which emulsified with Al(OH)_3_ on days 1, 7, and 14 except for the Naïve group. Immunotherapy was conducted continuously on days from 21 to 42. The mice were fed according to the following group arrangement. Naïve: the entire experiment was done without any manipulation used as a reference for physiological state and environmental variables. Saline: mice fed with saline 100 µl/ mouse/ day after sensitization. 1217A: fed with 2.5 mg/ 00 µl/mouse/ day modified LWDHW-1217A after sensitization. 1217B: fed with 2.5 mg/100 µl/mouse/day modified LWDHW-1217B. DEX: fed with Dexamethasone 1 µg/100 µl/mouse/day. AHR: Airway hyperresponsiveness; Sac: Sacrifice after the AHR measurement on day 46

The experimental procedures and operation methods of the asthmatic animal model mainly referred to our previous study [15]. Mice were sensitized three times by intraperitoneal (IP) injection with 10 µg Der p 2 emulsified in 4 mg aluminum hydroxide on day 1, day 7, and day 14 (Sigma-Aldrich, St. Louis, MO, USA) (Fig. 1). After 2 weeks of immunization, mice were continuously fed from day 21 to day 42 according to group arrangement, including fed with dexamethasone (DEX) (1 µg/100 µl/mouse), 1217A (2.5 mg/100 µl/mouse), 1217B (2.5 mg/100 µl/mouse) or normal saline 100 µl/mouse. Next, the mice were subjected to intratracheal (IT) challenge twice with nDer p 2 (5 µg/50 µl) on day 43 and day 44, and pulmonary function was measured by airway hyperresponsiveness (AHR) to methacholine on day 45. The naïve group did not receive any process entire experiment procedure. Blood and bronchoalveolar lavage fluid (BALF) were collected after the mice were sacrificed on day 46.

### Natural Der p 2 preparation

The natural Der p 2 (nDer p 2) was purchased from Indoor Biotechnologies, Incorporated (Virginia, United States of America). In brief, the nDer p 2 was purified from mite culture medium by affinity chromatography using an anti-mite Group 2 monoclonal antibody. The purity of nDer p 2 verified by SDS-PAGE was over 95%. The purified nDer p 2 was filtered through a 0.22 μm filter and conserved in phosphate-buffered saline (pH 7.4) under preservative-free and carrier-free conditions. The X-ray crystal structure and nuclear magnetic resonance structure of nDer p 2 had been confirmed and presented in the Protein Data Bank on the Website (https://www.rcsb.org/structure/1A9V). Avoid excessive endotoxin content to interfere with experimental data so the endotoxin detection kit was further analyzed. Endotoxin content in the purified nDer p 2 was detected by Pierce^™^ Chromogenic Endotoxin Quant Kit No. A39552 (Thermo Fisher Scientific^™^), this assay enables detection within linear sensitivity ranges of 0.01–0.1 EU/mL and 0.1–1.0 EU/mL. Endotoxin levels were determined by estimating the activity of Factor C in the presence of a synthetic peptide substrate that releases p-nitroaniline (pNA) after proteolysis, producing a yellow color that can be measured at an absorbance of 405 nm.

### Formula preparation of modified LWDHW- 1217A and 1217B

The formula of 1217A and 1217B are majorly referred to the Traditional Chinese Medicine known as Liu-Wei-Di-Huang Wan [26]. These two formulated herbal extracts of modified Liu-Wei-Di-Huang Wan (modified LWDHW), 1217A and 1217B, were prepared by Biomedical Engineering Center Industrial Technology Research Institute (Hsinchu, Taiwan). There were three herbs were selected adding to the formula of 1217A and 1217B in this study, including *Radix Dioscorea, Rhizoma Alismatis, and Scutellaria baicalensis,* based on their properties of anti-inflammatory and immunomodulatory effects on the allergic responses majorly referred other research findings. The formula of 1217A is composed of the traditional LWDHW and *Poria cocos* (ratio: 4)*, Radix Dioscorea* (Yam) (ratio: 3)*, Rhizoma Alismatis* (ratio: 1)*, and Scutellaria baicalensis* (ratio: 3) listed in Table 1. The difference between the formula of 1217A and 1217B is the composition of *Rhizoma Alismatis from* (3 g; ratio: 1) changed into (9 g; ratio: 3) so that the 1217B is composed of the traditional LWDHW and *Poria cocos* (ratio: 4)*, Radix Dioscorea* (Yam) (ratio: 3)*, Rhizoma Alismatis* (ratio: 3)*, and Scutellaria baicalensis* (ratio: 3) listed in Table 1. A total of 0.99 kg Chinese herbal medicine of 1217A and 1.05 kg of 1217B (the contents and ratios listed in Table 1) were extracted with a standard boiling protocol with 1L sterile water then filtered and lyophilized as herbal extract powder [26]. The lyophilized powders of herbal extracts (1217A and1217B) were saved at −20 ℃ and dissolved with normal saline before administrating to mice.

**Table 1:** The Composition and Ratio of Herbs Used in the Preparation of 1217A and 1217B from traditional Liu-Wei-Di-Huang-Wan

Traditional medicine	LWDHW		1217A		1217B	
Composition	Weight (g)	Ratio	Weight (g)	Ratio	Weight(g)	Ratio
*Rehmannia glutinosa*	24	8	24	8	24	8
*Cornus officinalis*	12	4	12	4	12	4
*Dioscorea opposita*	12	4	12	4	12	4
*Alisma orientalis*	9	3	9	3	9	3
*Poria cocos*	9	3	12	4	12	4
*Paeonia suffruticosa*	9	3	9	3	9	3
*Radix Dioscorea* (Yam)			9	3	9	3
*Alismatis Rhizoma*			3	1	9	3
*Scutellaria baicalensis* (Skullcap)			9	3	9	3

### High-performance liquid chromatograph (HPLC) situation

The HPLC analysis was conducted by a Waters Alliance e2695 separation Module and a waters 2996 photodiode assay detector coupled with a Millennium32^™^ Chromatography Manager Version 3.2 software. The separation was performed with a Waters Symmetry Shield RP18 column (MS C_18_ 4.6 × 250 mm, IS^™^ 5 µm at 25 ℃) coupled with a NOVA-Pak^™^ guard column. The mobile phase consisted of acetonitrile as A and 0.15% phosphoric acid water solution as B. The gradient elution was performed as follows: 10–20% acetonitrile for 0–15 min, 20–35% acetonitrile for 15–25 min, 35–100% acetonitrile for 25–45 min, and 100% acetonitrile for 45–50 min. The flow rate was set up as 1 ml/minute, and the column temperature was set at 30 ℃. The injection volume was set as 100 μl, and the detection wavelength was set at a full wavelength of 200–400 nm for the Chinese Medicine formula of 1217A and 1217B. The detect wavelengths of different standards were respectively according to the reference wavelength.

### Determination of allergen-specific immunoglobins in sera

Blood was obtained from the retro-orbital venous plexus and isolated using Ficoll-Paque Premium density gradient media (GE Healthcare) at the experiment’s beginning, checking point, and end. Serum level of Der p 2-specific IgE, IgG1, and IgG2a titers was determined by enzyme-linked immunosorbent assay (ELISA). The Der p 2 were coated in the 96-well microplates (Nunc Lab, IL, USA) at a concentration of 5 µg/ml with a coating buffer (0.05 M carbonate/bicarbonate; pH 9.6) with 100 µl volume at 4 °C overnight. Plates were washed with PBS-Tween-20 (PBST) three times and stored at 4 ℃ before use. Diluted mouse sera (1:5 dilution for IgE, 1:100 dilution for IgG1, and 1:25 dilution for IgG2a) were incubated and washed with PBS/0.05% Tween-20. The goat anti-mouse horseradish peroxidase-conjugated antibody (1:800 dilution for IgE and 1:2000 dilution for IgG1 and IgG2a) (Southern Biotech Assoc., Inc., Birmingham, AL, USA) was added. After incubating for one hour at room temperature, following washed three times with PBST, the color was developed with enzyme–substrate 3,3′,5,5′-tetramethylbenzidine (BioLegend, London, UK). The reaction was stopped with 50 µl H_2_SO_4_ (4N) and the optical density was measured at 450 nm in a multiscan spectrophotometer (model A-5682, SLT Lab Instruments, Salzburg, Austria). Results were expressed as ELISA units.

### Bronchoalveolar lavage fluid (BALF) collection and Cytokine Measurements

Two separate injections of 1 ml sterile endotoxin-free saline were injected into the mouse lungs via trachea for receiving BALF. Approximately 1.8 ml of the BALF was recovered constantly. The BALFs were respectively collected and stored at −70 ℃. The cytokines IL-5, IL-13, IFN-γ, and IL-12 were measured using commercial ELISA kits (R& D Systems, Minneapolis, MN, USA) containing monoclonal antibodies (mAbs) against different cytokines. The detailed procedures were conducted according to the manufacturer’s instructions. The results of cytokines in BALF were presented as pg/mL and the lowest detectable concentration was 10 pg/mL.

### Inflammatory cell counting by cytospin smears

A total of 100 µl BALF was performed by cytospin preparation through a cytocentrifugation process for leukocyte counting. The BALF was loaded in special plastic sample chambers, centrifuged at 1,500 rpm, and the cells were deposited onto a vertical microscope slide. The suspended BALF was absorbed by the filter and the cells were sedimented within a 32 mm area of the glass slide. The cells were stained with Liu’s stain (Tonyar Diagnostic Inc., New Taipei City, Taiwan), and the data was presented as 10^4^/mL in BALF of the specific leukocyte numbers.

### Pulmonary function measurement

The pulmonary function of mice was measured by a whole-body plethysmograph system (Buxco Electronics, NY, USA) with two individual chambers as described previously [15]. Mice were placed inside a barometric plethysmograph, which the plethysmograph has two chambers: one is the main or animal chamber and the other is the reference chamber. The pressure signal was amplified, digitized via an A/D convert card, and sent to a computer with a BioSystem XA program (Buxco, Electronics, Troy, NY). Parameters of enhanced pause (Penh), pause, tidal volume (V_T_), breathing frequency (f), peak inspiratory flow (PTF), peak expiratory flow (PEF), end-inspiratory pause (EIP), and end-expiratory pause (EEP) were recorded. Aerosol was generated by placing PBS or methacholine (6.25, 12.5, and 25.00 mg/ml) solution in the cup of an ultrasonic nebulizer (DeVilbiss, Somerset, PA) and it was delivered via connecting tube and three-way connector to the animal chamber of the plethysmograph. Each mouse inhaled saline aerosol for 3 min and then the respiratory parameters were measured for 3 min. The aerosol in the chamber was cleared immediately after the exposure. Respiratory parameters were then measured for 3 min following the inhalation of the methacholine aerosol. Pulmonary resistance was expressed as enhanced pause (Penh). The Penh values of different groups were obtained from each mouse induced with a distinct dose of PBS or methacholine and were presented as mean ± standard error of the mean (SEM).

### Cell culture, immunofluorescence staining, Th1/ Th2 cytokine stimulation, and flow cytometry

Blood samples were acquired with a heparin tube and isolated the peripheral blood mononuclear cells (PBMC) by density centrifugation using Ficoll-Paque Plus density gradient for cell separation and culture. Leukocytes from the PBMC of six mice were pooled for the purpose of collecting enough cells. Then, the cells were stimulated with phorbol myristate acetate (PMA; 50 ng/ml), ionomycin (2 μmol/L), and GolgiStop (Cytofix/Cytoperm Plus PharMingen, San Diego, CA, USA) for 5 h resulting in T cell activation and cytokine productions and then washed twice with PBS. The immunofluorescence staining was performed by three-color staining procedures to analyze the expression of Th1-type cytokine (IFN-γ) and Th2-type cytokine (IL-4) in the CD4^+^ [15]. The cells were stained with peridinin chlorophyll protein-conjugated rat anti-mouse CD4 mAb (BD, Biosciences) at room temperature for 30 min. Then, cells were fixed with cytofix/cytoperm and washed with PBS. Cells stained with fluorescein isothiocyanate (FITC)-conjugated rat anti-mouse IFN-γ mAb (BD, Biosciences) and R-phycoerythrin-conjugated rat anti-mouse IL-4 mAb (BD, Biosciences) at room temperature. After being washed, cells were resuspended in 0.5 ml PBS with 0.1% sodium azide. Mean fluorescence was measured by BD FACSCalibur™ flow cytometry (Becton Dickinson, San Jose, CA, USA). A total of 1 × 10^4^ cells were analyzed for each sample. The flow cytometer was equipped with 488 nm and 633 nm lasers capable of detecting and distinguishing fluorescence emissions at 430 nm and 685 nm. Quantifications of cytokines were demonstrated by a flow cytometry scatter plot. The flow data were performed in three independent replicates of experiments, and data were presented as the mean ± SD of three independent experiments.

### Cell viability assay by flow cytometry

Cell viability is used to measure the percentage of living cells after treatment and evaluated by observing changes in membrane permeability or physiological state with the expression of vital dyes. The Propidium iodide (PI) dye (Sigma-Aldrich) was used as a fluorescent nucleic-acid dye that penetrates the cell membrane and binds to DNA, causing the cell to emit red fluorescence and detected by flow cytometry. Flow cytometry assays were performed in triplicate and fluorescence emission was with an excitation laser line of 493 nm and maximum emission at 632 nm.

### Phosphorylation of STAT6, STAT1, and ERK and activation of GATA3 by Western blot analysis

The lung from the individual mouse of each group was collected, homogenized, and lysed in PRO-PRER^™^ protein extraction solution (iNtRON Biotechnology, Seoul, South Korea). The progress of this experiment was performed with each individual mouse to confirm the effects of TCM on the signaling pathway, not with pooled combination between group. There were six mice conducted from each group. Data presented were obtained from at least three independent experiments. Protein concentration was determined by Bradford assay (Bio-Rad, Hercules, Calif., USA) using bovine albumin as a standard. The protein concentrations of samples were obtained by program calculation (GraphPad Prism 8). Protein samples were subjected to separate on 12% of sodium dodecyl sulfate–polyacrylamide gel electrophoresis (SDS-PAGE) and transferred to polyvinylidene difluoride (PVDF) membrane. The membrane was blocked with 5% skim milk and washed three times with PBST (PBS containing 1% Tween-20). The membrane was incubated with primary antibodies at appropriate dilution with gentle agitation at 4 ℃ overnight, followed by incubation with horseradish peroxidase-coupled secondary antibody (catalog No. AP308P) (1:3000 dilution, Sigma-Aldrich, St. Louis, Mo., USA). The primary antibodies used were as follows: rabbit anti-mouse phosphate-STAT6 (Y641) (1:1000 dilution, R&D Systems, Minneapolis, MN, USA); rabbit anti-mouse phosphorylated -ERK (1:2000 dilution, R&D Systems); rabbit anti-mouse GATA-3 (1:3000 dilution, Thermo Fisher Scientific Inc. MA., USA); rabbit anti-mouse phosphorylated-STAT1(Y701) (1:1000 dilution, R&D Systems); rabbit anti-mouse STAT6 (Cat No. #9362) (1:1000 dilution, Cell Signaling Technology); and rabbit anti-mouse STAT1 (Cat No. #9172) (1:1000 dilution, Cell Signaling Technology). The primary antibodies and secondary antibodies were diluted with PBS. Signals were visualized by enhanced chemiluminescence (ECL) and Amersham Hyperfilm ECL (GE Healthcare Life Science, Chicago, IL., USA). The Western blot signals were quantified by VisionWorks LS software of UVP BioSpectrum^™^ 500 Imaging System (Thermo Fisher Scientific Inc. Waltham, MA., USA).

### Pathology of lung trachea tissue

The lung and trachea tissues of mice from different groups were cut off, washed out with PBS, and infiltrated in 10% formalin after the animals had been sacrificed. The serial sections of lung tissue were split at 5 μm thickness and stained with hematoxylin–eosin. These tissue sections on the slides were mounted with a coverslip. The histologic scores of lung pathology for each field were following tracheal thickness, inflammatory cell count, and tracheal rupture. Ten visual fields of each mouse were examined, and the differences among groups in histologic scores were analyzed.

### Statistical analysis

Statistical analyses were carried out using SPSS, Version 12 (SPSS, Inc., Chicago, IL, USA). Sample Power 2.0 was used for power calculation analysis. The differences between groups were analyzed with Mann–Whitney U-test for non-parametric analysis. The diagrams of statistical analyses were performed using GraphPad Prism 5 (GraphPad Software, San Diego, CA, USA). Data are presented as mean ± deviation (SD). A value of *p* < 0.05 was considered statistically significant.

## Results

### Qualitative analysis of representative standard compounds in modified LWDHW-1217A and 1217B

**Fig. 2:**
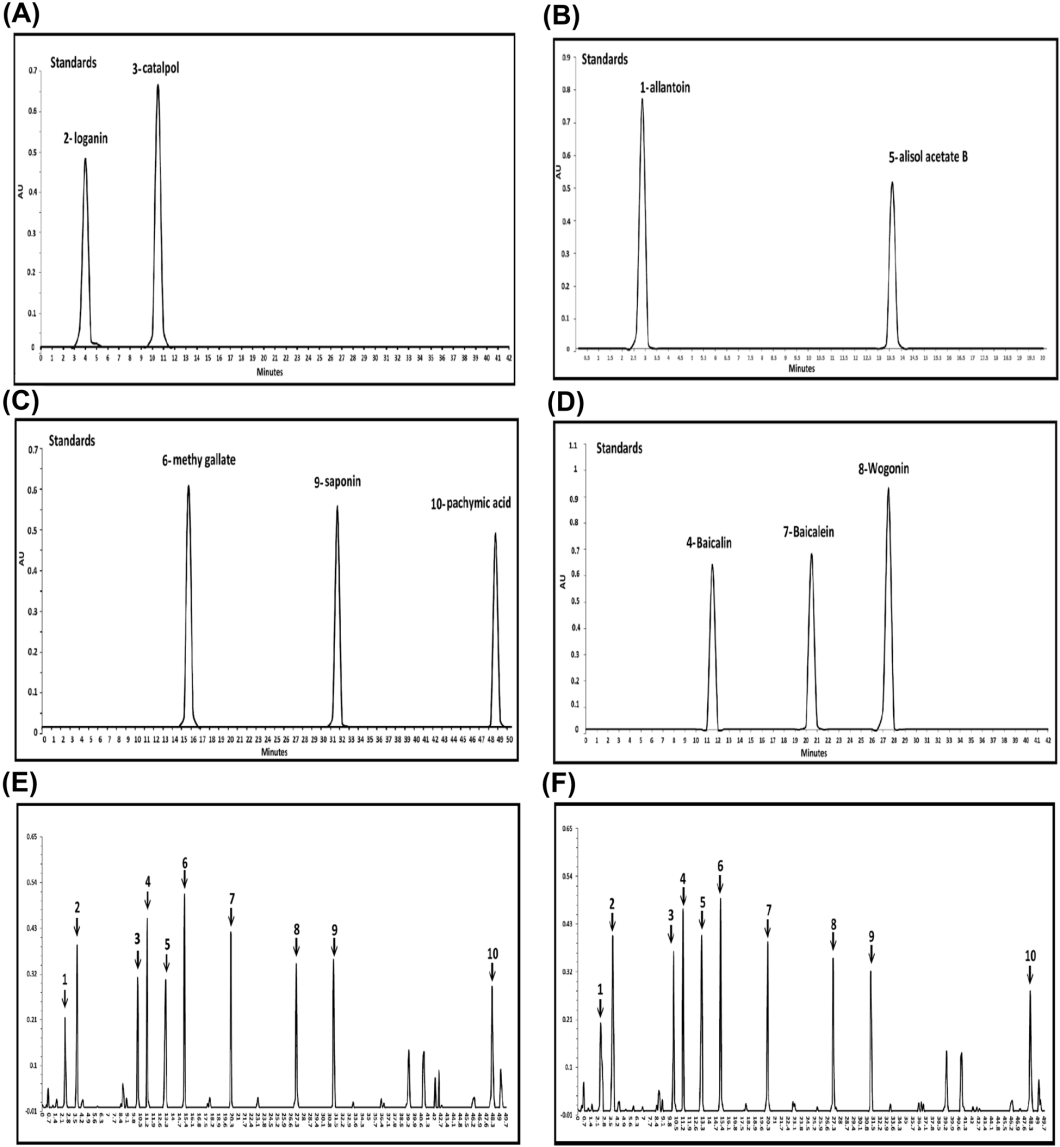
HPLC chromatograms of ten representative standards and formula of TCM-1217A or 1217B. **A** Retention time of standard- loganin and catalpol. **B** Retention time of standard-allantoin and alisol acetate B. **C** Retention time of standard- pachymic acid, methyl gallate, and saponin. **D** Retention time of standard- baicalin, baicalein, and wogonin. **E** Retention time of formula TCM-1217A. **F** Retention time of formula TCM-1217B

The major active ingredients in modified LWDHW-1217A and 1217B were identified by HPLC analysis. The active compound of catalpol in *Rehmannia glutinosa* and loganin in *Cornus officinalis* were used as standards, which their retention time were at 10.5 min and 4.0 min, respectively (Fig. 2A). The active ingredient of allantoin in *Dioscorea opposite* and alisol acetate B in *Alisma orientalis* were listed*,* their retention time of these standards were at 2.75 min and 13.5 min (Fig. 2B). The retention time of active compound-pachymic acid in *Poria cocos* was at 48.5-min*,* the retention time of active compound-methyl gallate in *Paeonia suffruticosa* was at 15.5-min*,* and the saponin in *Radix Dioscorea* was at 31.5-min (Fig. 2C). These three active compounds of baicalin, baicalein, and wogonin in *Scutellaria baicalensis* were listed, their retention time of these standards was at 11.5 min, 20.5 min, and 27.5 min, respectively (Fig. 2D). In the designed chromatogram condition, the primary active compounds were identified in the formula of modified LWDHW- 1217A and 1217B that contained at least ten active ingredients order by the retention time including 1-allantoin, 2-loganin, 3-catalpol, 4-baicalin, 5- alisol acetate B, 6-methyl gallate, 7-baicalein, 8-wogonin, 9-saponin, and 10-pachymic acid (Fig. 2E and F). The main difference between the formula of modified LWDHW-1217A and 1217B was the active ingredient of 5-alisol acetate B from *Alismatis Rhizoma* at a retention time of 13.5 (Fig. 2F).

### Effects of modified LWDHW-1217A and 1217B on the productions of specific IgG1, IgG2a, and IgE in the sera of Der p 2-sensitized mice

**Fig. 3:**
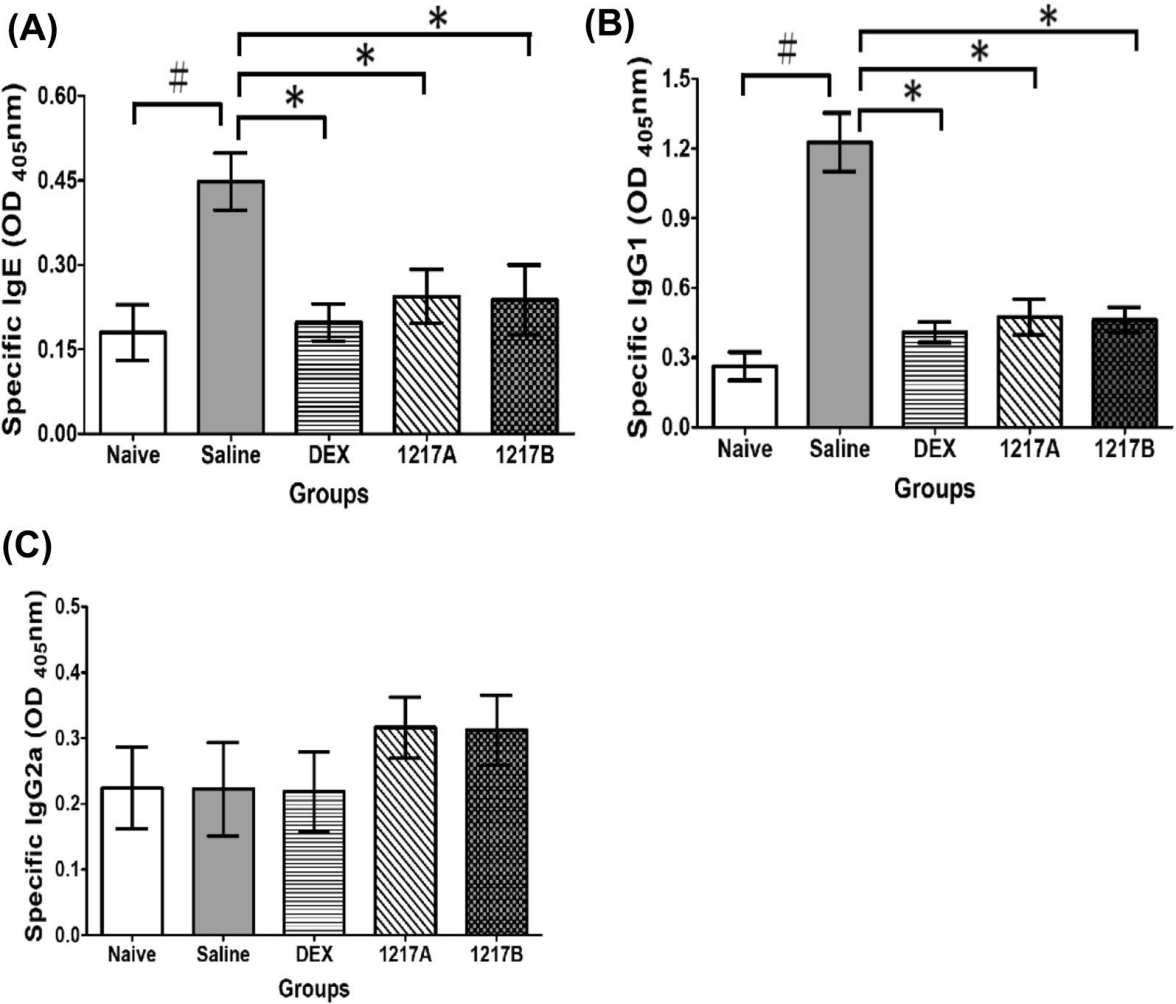
Productions of Der p 2-specific IgE, IgG1, and IgG2a in the sera from different groups after TCM-1217A or 1217B treatment. Serum concentrations of Der p 2-specific IgE, IgG1, and IgG2a on Day 46 were measured by ELISA after mice had been sacrificed. **A** Der p 2-specific IgE, **B** Der p 2-specific IgG1, **C** Der p 2-specific IgG2a. Values were presented as means ± SD of optical density (OD) 405 nm of each group (*n* = 6). #: Compared to the Naive group; *: *p* < 0.05 compared to the immunotherapy with the saline group; 1217A: immunotherapy with TCM of modified LWDHW-1217A; 1217B immunotherapy with TCM of modified LWDHW-1217B

Before the animal experiments, the endotoxin content in the purified nDer p 2 was examined in triplicate and the value was 0.15 ± 0.01 EU/ml. To determine the effect of 1217A and 1217B on immunoglobulin production, the mite allergen nDer p 2 was used for intraperitoneal sensitization. The Der p 2-specific IgE, IgG1, and IgG2a in the sera were measured after immunotherapy with 1217A or 1217B. After the IP sensitization and immunotherapy with saline, the Der p 2-specific IgE and IgG1 were significantly higher in the sera when compared with the naive group (Fig. 3A; *p* < 0.01 and Fig. 3B; *p* < 0.01). The differences between groups were analyzed with Mann–Whitney U-test for non-parametric analysis. Data are presented as mean ± SD and the *p* < 0.05 was considered statistically significant. In the immunotherapy with 1217A or 1217B, the Der p 2-specific IgE and IgG1 were significantly reduced when compared to that of the saline group (Fig. 3A and B; *p* < 0.05). However, there were no relevant changes in IgG2a compared between the naïve group and the saline group, the same as in the comparison between the saline group and other therapeutic groups (Fig. 3C). Results indicated that the productions of mite allergen-specific IgE and IgG1 could be down-regulated after immunotherapy with 1217A or 1217B in the mite-sensitized mice.

### Effects of modified LWDHW-1217A and 1217B on the productions of cytokines in the Sera and BALF of Der p 2-sensitized mice

**Fig. 4:**
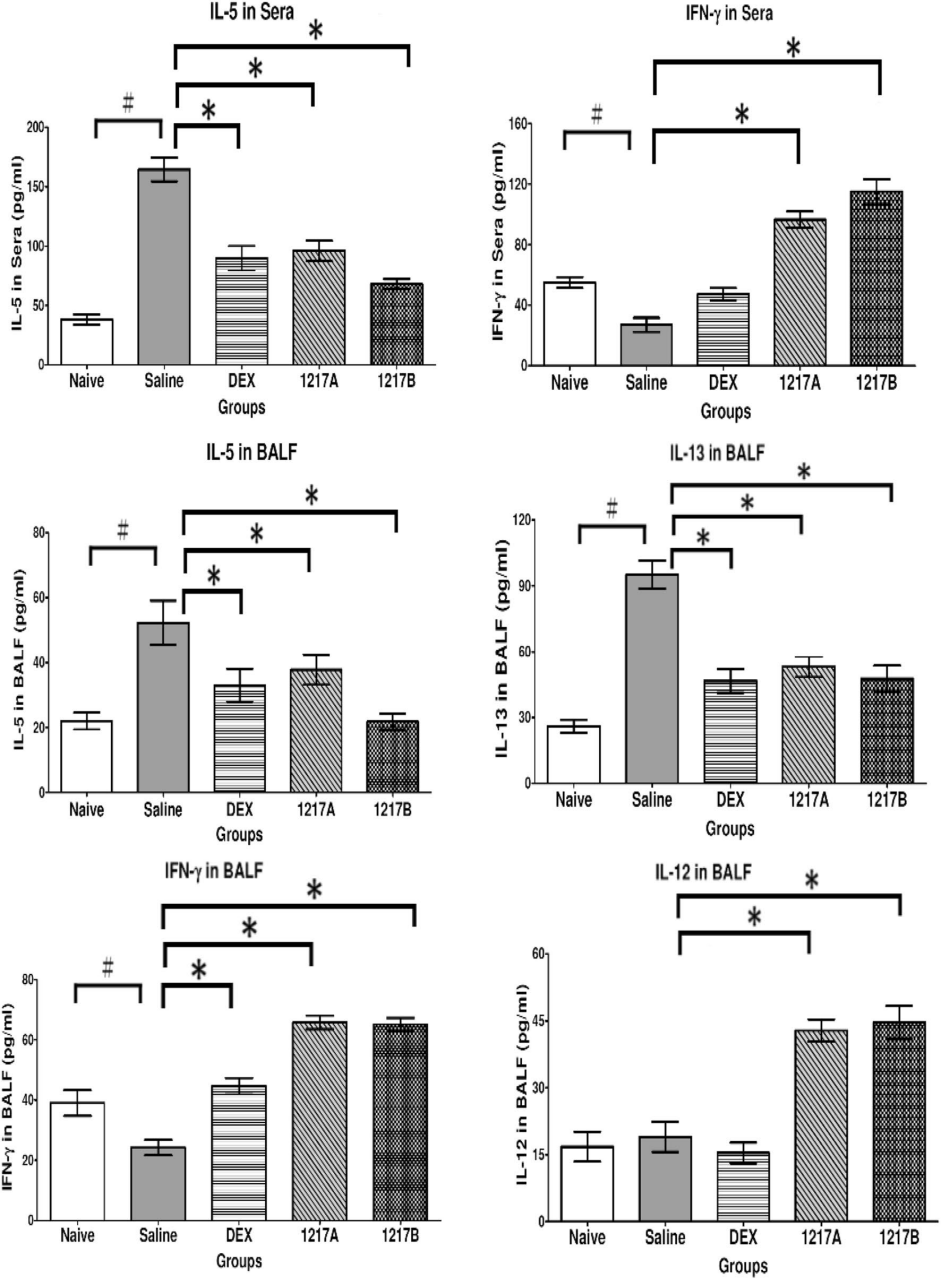
Differences in inflammatory cytokine or Th1-related cytokine productions in sera and BALF were measured by ELISA after immunotherapy. The cytokine productions of IL-5 and IFN-γ in the sera were measured. The BALFs were collected from mice after sacrifice and cytokine productions of IL-5 and IFN-γ in the sera were measured. Data for each cell are expressed as means ± SD pg/mL for each group (*n* = 6). #: *p* < 0.05 compared to the Naive group; *: *p* < 0.05 compared to the treatment with saline. The groups meant the use of agents during immunotherapy after the allergen sensitization

To determine the effect of modified LWDHW*-*1217A or 1217B on cytokine productions, the cytokines in the sera and BALF were measured by ELISA after immunotherapy with 1217A or 1217B. In the group of mice treated with IP sensitization and immunotherapy with saline, the levels of inflammatory cytokine of IL-5 in the sera and BLAF, and the IL-13 in the BALF were significantly increased compared with the naïve control (Fig. 4; *p* < 0.05). When the mice were treated with modified LWDHW*-*1217A or1217B after the allergen Der p 2 IP sensitization, the results showed there were significant reductions of inflammatory cytokine IL-5 in sera and BALF and the IL-13 in the BALF; whereas, the Th1-cytokine IFN-γ in sera, and the IL-12 and IFN-γ in the BALF were significantly increased in comparison with the saline group (Fig. 4, *p* < 0.05). The inhibitory effects of the inflammatory cytokine IL-5 in the sera and inflammatory cytokines of IL-5 and IL-13 in BALF have also been observed in the mice treated with Dexamethasone. Although there was also a significant change in Th2-cytokines (IL-5 and IL-13) in the group treated with Dexamethasone, it did not increase Th1 cytokines. The immunotherapy with modified LWDHW-1217A or 1217B was also able to cause a decrease in inflammatory cytokines (IL-5 and IL-13) and an increase in Th1-cytokine (IL-12 and IFN-γ) in the airway BALF. Results indicated the Modified LWDHW-1217A or 1217B has the potential to down-regulate the inflammatory tendency caused by the mite allergen Der p 2-induced asthmatic model. Overall, the Th2 inhibitory effect of 1217B is slightly higher than that of 1217A, and the effect of activating Th1 of 1217B is also slightly higher than that of 1217A.

### Effects of modified LWDHW-1217A and 1217B on the inflammatory cell infiltrations in the airway of Der p 2-sensitized mice

**Fig. 5:**
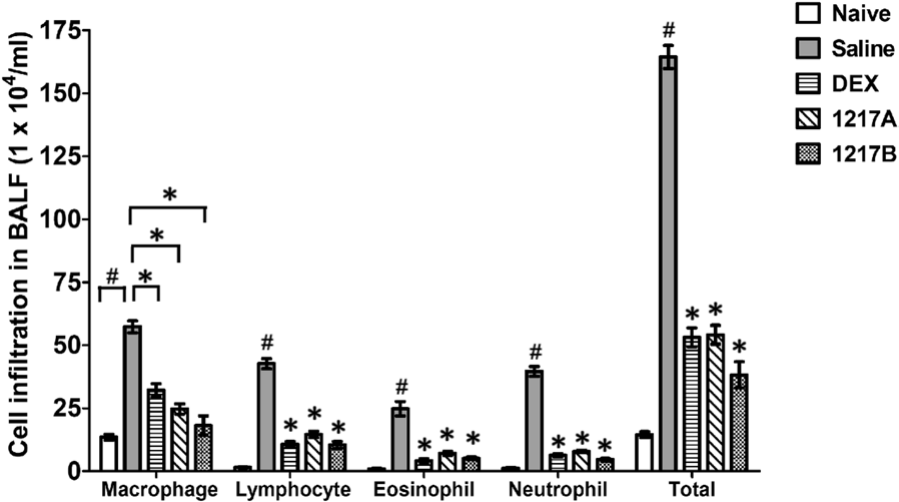
Effects of 1217A and 1217B on inflammatory cell infiltration in the airway. Differences of inflammatory leukocyte subpopulations in BALF from different groups after sacrifice. Inflammatory cell infiltrations of leukocyte subpopulations including macrophages, lymphocytes, eosinophils, and neutrophils in BALF were measured by Cytospin smears for leukocyte counting. Total: total cells of leukocytes. Data for each cell are expressed as means ± SD × 10^4^ cells/mL for each group (*n* = 6). #:* p* < 0.05 compared to the Naive group; *: *p* < 0.05 compared to the immunotherapy with the saline group; Groups meant the usage during the immunotherapy

The inflammatory cell infiltrations in the airway were compared in all experimental groups of mice by analyzing the leukocytes subpopulation in the BALF. After the IP sensitization with mite allergen Der p 2, the mice were immunotherapy with different experimental designs. Next, the mice were IT challenged with Der p 2 twice after the therapy, and airway inflammatory cell infiltration was collected and analyzed. After the allergen challenge, the airway infiltrations of inflammatory cells including macrophages, eosinophils, neutrophils, and lymphocytes were significantly increased in the group of mice that IP sensitization with mite allergen followed by immunotherapy with saline when compared to the naïve group (Fig. 5; *p* < 0.05). There were significant reductions in inflammatory cell infiltrations including macrophages, eosinophils, neutrophils, and lymphocytes after immunotherapy with modified LWDHW-1217A or 1217B in comparison with the saline group (Fig. 5; *p* < 0.05). There was also a significant decrease in the total cell numbers in mice treated with 1217A, 1217B, or Dexamethasone in comparison with the saline group (Fig. 5; *p* < 0.05). Results indicated that immunotherapy with modified LWDHW-1217A or 1217B had anti-inflammatory effects on inflammatory cell infiltration in the airway, especially in 1217B has better inhibitory effects.

### Effects of modified LWDHW-1217A and 1217B on the gene expressions in lung tissue of Der p 2-sensitized mice

**Fig. 6:**
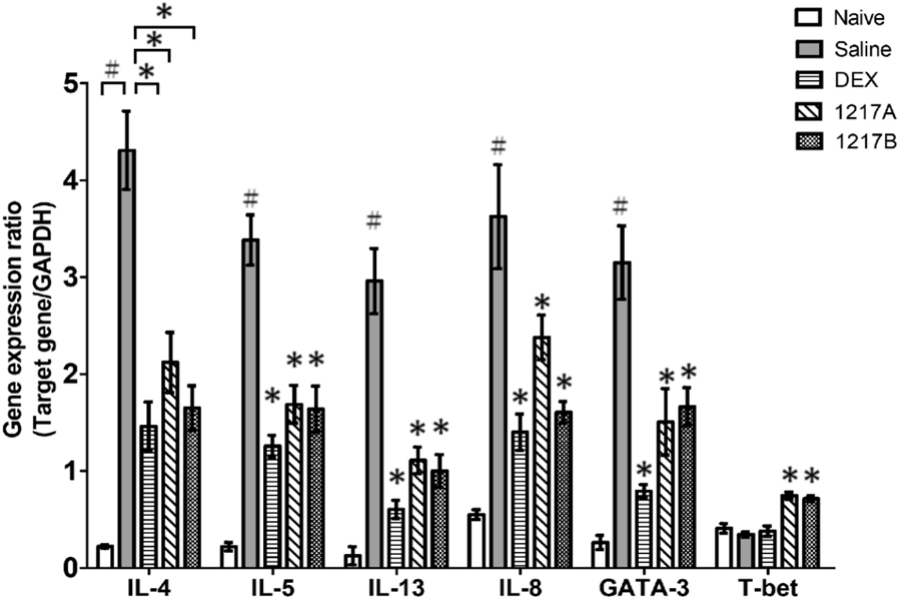
Effects of 1217A and 1217B on the Gene expressions of the inflammatory cytokines, chemokine, and transcription factors in lung tissues. Total mRNA was extracted from the lung tissue of mice after sacrifice. The mRNA of lung tissue was acquired for real-time quantitative-PCR assay (Q-PCR) to investigate gene expressions. The T_H_2-related genes (*IL-4*, *IL-5*, and *IL-13*), T_H_2-related transcription factor (*GATA-3*), neutrophil chemotactic chemokine (*IL-8*), and the T_H_1-related transcription factor (*T-bet*) were used to evaluate the gene variations. GAPDH is used as an internal control gene for normalizing the target gene expression of each sample. The histogram shows the normalized expression levels of the target gene/ *GAPDH* as mean ± SD. #:* p* < 0.05 compared to the Naive group; *: *p* < 0.05 compared to the treatment with saline

The lung tissue from mice was acquired for real-time quantitative-PCR assay (Q-PCR) to investigate gene expressions related to allergy after the IP sensitization, immunotherapy with Dexamethasone, *Modified LWDHW*, and IT challenges with mite allergen. To assess gene alternations in mite-induced asthmatic mice following immunotherapy with modified LWDHW1217A or 1217B, the expression levels of TH2-related genes including *IL-4, IL-5*, and *IL-13*, TH2-related transcription factor GATA-3, neutrophil chemotactic chemokine IL-8, and TH1-related transcription factor T-bet were analyzed. GAPDH was used as the control gene for normalizing the target gene expression of each sample. After the IP sensitization of mite allergen and immunotherapy with saline, the Q-PCR results revealed the T_H_2-related genes of *IL-4*, *IL-5*, and *IL-13* (4.4-fold, 3.5-fold, and 3.0-fold relative to baseline *GAP-DH*, respectively) were significant upregulation when compared to the naïve group (*p* < 0.05; Fig. 6). The group immunotherapy with saline also had a 3.8-fold increase in expression of neutrophil chemotactic chemokine (*IL-8*) (*p* < 0.05), and a 3.1-fold increase in expression of T_H_2-related transcription factor (*GATA-3*) (*p* < 0.05). In the group of mice treated with IP sensitization and immunotherapy with modified LWDHW1217A or 1217B, the T_H_2-related genes of *IL-4*, *IL-5*, and *IL-13*, the neutrophil chemotactic chemokine of *IL-8*, and T_H_2-related transcription factor of *GATA-3* were significantly decreased in the lung tissue when compared with the mice immunotherapy with saline (Fig. 6; *p* < 0.05). Similar findings were also observed that these T_H_2-related genes (*IL-4*, *IL-5*, and *IL-13*), chemokine gene (*IL-8*), and T_H_2-related transcription factor (*GATA-3*) were significantly down-regulated in the group of mice that received immunotherapy with Dexamethasone (Fig. 6; *p* < 0.05). Moreover, the T_H_1-related transcription factor (*T-bet*) was significantly increased in the group of mice immunotherapy with modified LWDHW1217A or 1217B in comparison with the saline group (Fig. 6;* p* < 0.05). Overall, immunotherapy modified LWDHW1217A or 1217B was also able to cause a decrease in expressions of inflammatory genes (*IL-4*, *IL-5*, and *IL-13*), neutrophil chemotactic chemokine (*IL-8*), and T_H_2-related transcription factor (*GATA-3*); on the other hand, an increase in the T_H_1-related transcription factor (*T-bet*) in the lung tissue of asthmatic mice.

### Effects of modified LWDHW-1217A and 1217B on the phosphorylation of STAT6, STAT1, and ERK and activation of GATA3

**Fig. 7:**
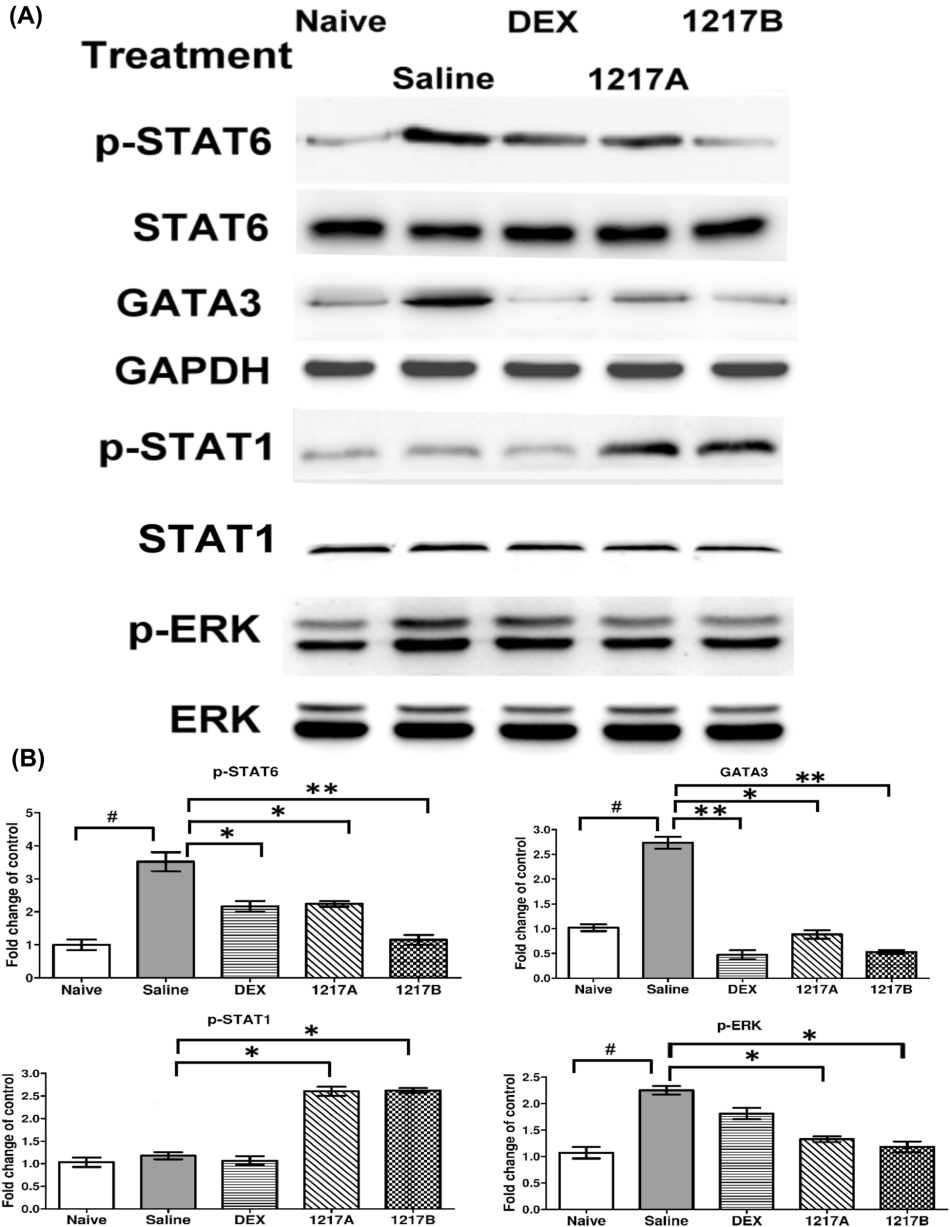
Effects of TCM-1217A and 1217B on the phosphorylation of STAT6, STAT1, and ERK and activation of GATA3. Protein samples from lung tissue lysates among the groups were separated on 12% of SDS-PAGE and transferred to PVDF membranes. Equal amounts of proteins were used for Western blotting using GAPDH, STAT1, and STAT6 as an internal control of protein levels among groups. There were six mice conducted from each group. Representative data showed according to their treatment. All experiments were performed at least three times. **A** The representative data of phosphorylated(p)-STAT6, GATA3, p-STAT1, and p-ERK among different groups with individual treatment, as indicated in the panel. **B** The relative levels of p-STAT6, GATA3, p-STAT1, and p-ERK are presented as mean ± SD with a histogram. #:* p* < 0.05 compared to the Naive group; *: *p* < 0.05 compared to the saline group. **: *p* < 0.01 compared to the saline group

The STAT6 is mainly activated by cytokines including IL-4 and IL-13. The STAT6-mediated signaling pathway is required for the development of T-helper type 2 (Th2) cells and Th2 immune response. STAT6 plays a critical role in Th2 lung inflammatory responses and the pathogenesis of asthma. STAT1 can be activated by several ligands like IFN-γ, and then the IFN-γ signaling has been further shown to inhibit IL-4 expression by inhibiting STAT6 phosphorylation. The STAT1 signaling mechanism upregulates T-bet and mediates repression of *IL-4* gene in T_H_1 cells that subsequently represses GATA3 and IL-4 expression and antagonizes the STAT6 signaling. The ERK signaling activation participates in the release of the inflammatory mediators. The anti-inflammatory mechanisms of modified LWDHW-1217A and 1217B on the phosphorylation of STAT6, STAT1, and ERK and activation of GATA3 were investigated in the following experiment. After the IP sensitization and immunotherapy with saline, the phosphorylation of STAT6 and ERK and the expression of GATA3 were significantly enhanced compared with the naïve group (Fig. 7;* p* < 0.05). The expression levels of total ERK protein and GAPDH showed very similar among all groups. After immunotherapy with modified LWDHW1217A or 1217B, the expressions of phosphorylated-STAT6, phosphorylated-ERK, and GATA3 were significantly decreased when compared to the group which immunotherapy with saline (Fig. 7; **:*p* < 0.01; *:* p* < 0.05). The inhibitory effects of phosphorylated-STAT6, phosphorylated-ERK, and GATA3 could be also shown in the immunotherapy with Dexamethasone (Fig. 7; *p* < 0.05). The phosphorylation of T_H_1-related transcription factor- STAT1- was significantly increased in the group immunotherapy with modified LWDHW-1217A or 1217B when compared to the saline group (Fig. 7; *p* < 0.05). The phosphorylation of STAT1 seemed not to change significantly after immunotherapy with Dexamethasone when compared with those of saline (Fig. 7; *p* < 0.05). Results indicated that immunotherapy with modified LWDHW1217A or 1217B can enhance the T_H_1 cell-related activities or responses and suppress the T_H_2 cell-related activities or responses. However, the T_H_1 cell-related and T_H_2 cell-related responses were both suppressed in the group immunotherapy with Dexamethasone.

### Effects of modified LWDHW-1217A and 1217B on the cell percentages in CD3^−^/CD4^+^/CD8^+^ and Th1/Th2 cytokine expression in CD4 + Leukocytes in the Der p 2-sensitized mice

**Fig. 8:**
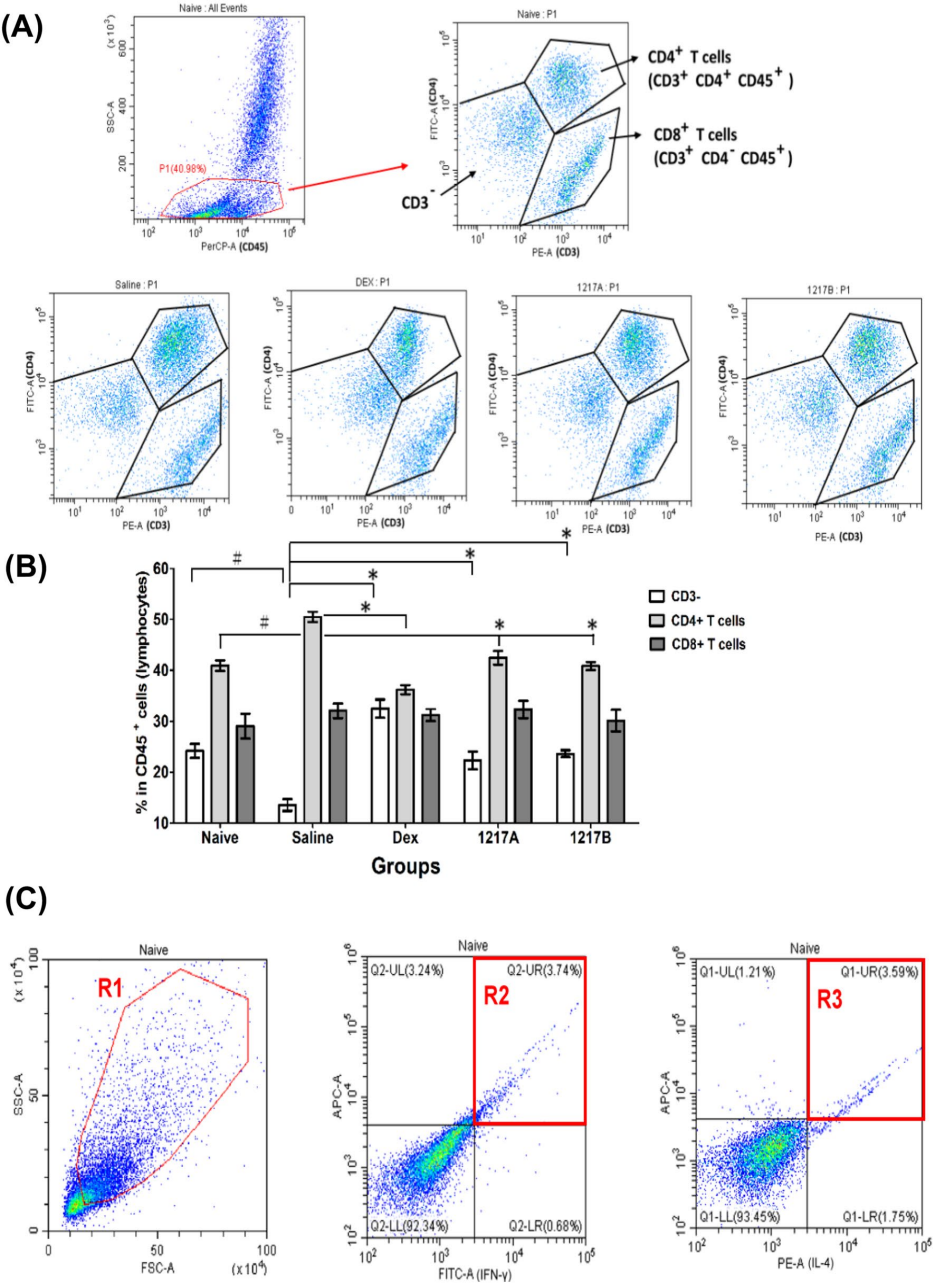

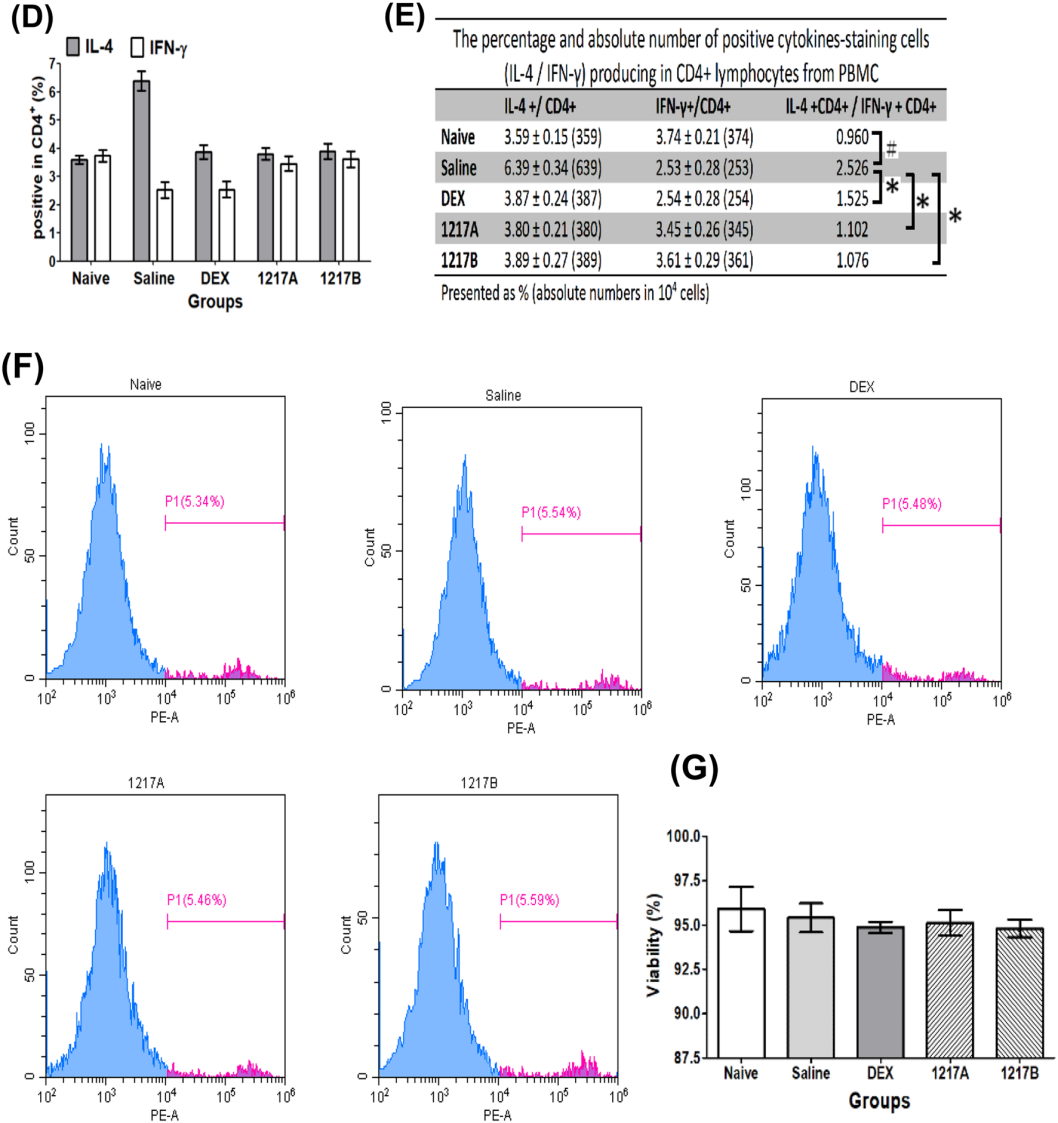
The flow cytometry analysis of cell percentages in CD3^−^/CD4^+^/CD8^+^ and Th1/Th2 cytokine expression in CD4^+^ leukocytes. **A** Three-color staining was used to analyze the expression of CD3^+^ CD4^+^ (CD4^+^ T cells) and CD3^+^ CD4^−^ (CD8^+^ T cells) in the CD45^+^ cells. The lymphocytes were gated on an SSC (side scatter) vs. CD45^+^ cells dot plot as gate region-P1 (P1). Three separate populations of CD45^+^ cells appeared in this three-color staining. The CD3^−^ expression cells are on the bottom left. Expressions of CD3^+^ CD4^+^ cells (CD4^+^ T cells) on the upper right and CD3^+^ CD4^−^ (CD8^+^ T cells) on the bottom right. **B** Data are presented as the mean ± SD of three independent experiments. A total of 1 × 10^4^ cells were analyzed for each sample. #: *p* < 0.05 compared to the Naïve group; *: *p* < 0.05 compared to the saline group. **C** Three-color staining was used to analyze the expression of Th1-type cytokine (IFN-γ) and Th2- type cytokine (IL-4) in the CD4^+^. Lymphocytes were gated on an FSC (forward scatter) vs. SSC (side scatter) dot plot as gate region-1 (R1). Expression of IFN-γ in CD4^+^ T cells gated as region-2 (R2) and IL-4 in CD4^+^ T cells gated as region-3 (R3). **D** Percentages of IFN-γ^+^/CD4^+^ and IL-4^+^/CD4^+^ T cells after immunotherapy present as a histogram of means ± SD. A total of 1 × 10^4^ cells were analyzed for each sample. **E** The expression portion of IFN-γ^+^/CD4^+^ and IL-4^+^/CD4^+^ T cells is presented as a percentage. The ratio of Th2/Th1 was calculated as IL-4^+^/CD4^+^ divided by IFN-γ^+^/CD4^+^. #: *p* < 0.05 compared to the Naïve group; *: *p* < 0.05 compared to the saline group. **F** Cell viability assay with propidium iodide staining by flow cytometry. Fluorescence greater than 10^4^ is regarded as dead cells and presented as P1. **G** Percentage of cell viability presented as mean ± SD in three independent repetitions

After immunotherapy with 1217A or 1217B*,* the peripheral blood leukocytes from mice were collected with three-color staining (CD45, CD3, and CD4) for realizing the CD4^+^ and CD8^+^ in the CD3^+^ cell population. After staining, the cell subpopulation and fluorescence quantification were analyzed by the flow cytometer. After the analysis, the lymphocytes were gated on an SSC (side scatter) vs. CD45^+^ cells dot plot as gate region-P1 (P1) (Fig. 8A). There were three separate cell populations appeared in CD45^+^ cells, respectively CD3^−^ cells on the bottom left, CD3^+^ CD4^+^ cells (CD4^+^ T cells) on the upper right, and CD3^+^ CD4^−^ (CD8^+^ T cells) on the bottom right (Fig. 8A). The results revealed that the CD3^−^ cells could be significantly downregulated and the CD4^+^ T cells could be significantly upregulated in the group immunotherapy with saline when compared to the naïve group; while CD8^+^ T cells no difference between these groups. After immunotherapy with modified LWDHW-1217A or 1217B*,* the percentage of CD4^+^ T cells was significantly downregulated when compared to the saline group, which the percentage of CD4^+^ T cells similar to the naïve group (Fig. 8B). After immunotherapy with the Dexamethasone, the percentage of CD4^+^ T cells was also apparently downregulated, and the CD3^−^ T cells significant increase when compared to the saline group (Fig. 8B). Since there were significantly different in the CD4^+^ T cells among these groups, further investigation focusing on the expression portion of the Th1-type cytokine (IFN-γ) and Th2-type cytokine (IL-4) producing on the CD4^+^ cells were analyzed. The lymphocytes were gated on an FSC (forward scatter) vs. SSC dot plot as gate region-1 (R1) shown in Fig. 8C. The expression of IFN-γ and IL-4 among the CD4^+^ T cells were gated as region-2 (R2) and region-3 (R3), respectively (Fig. 8C). The percentages and absolute numbers of IFN-γ^+^/CD4^+^ and IL-4^+^/CD4^+^ T cells after immunotherapy among different groups were compared and presented as a histogram (Fig. 8D and E). The results revealed that the IL-4^+^/CD4^+^ T cells (6.39 ± 0.34%) could be significantly upregulated and the IFN-γ^+^/CD4^+^ T cells (2.53 ± 0.28%) could be significantly downregulated in the group of mice immunotherapy with saline after Der p 2 sensitization when compared to the those of naïve group. The ratio of Th2/Th1 was 0.658 in the naïve group; furthermore, there were obvious changes in the Th2/Th1 ratio of the saline group (0.960) which was sensitized with Der p 2 mite allergen and immunotherapy with saline (Fig. 8E). After immunotherapy with modified LWDHW-1217A, the population of IL-4^+^/CD4^+^ T cells could be downregulated to 3.80 ± 0.21%, and the IFN-γ^+^/CD4^+^ T cells upregulated to 3.45 ± 0.26%, whereas the ratio of Th2/Th1 was 1.102 (Fig. 8E). Similar results can also be found in the immunotherapy with the modified LWDHW-1217B that the population of IL-4^+^/CD4^+^ T cells downregulated to 3.89 ± 0.27%, and the IFN-γ^+^/CD4^+^ T cells upregulated to 3.61 ± 0.29%, whereas the ratio of Th2/Th1 was 1.076 (Fig. 8E). After immunotherapy with the Dexamethasone, not only the cell population of IL-4^+^/CD4^+^ T cells was inhibited to 3.87 ± 0.24%, but also the IFN-γ^+^/CD4^+^ T cells was inhibited and decreased to 2.54 ± 0.28%, whereas the ratio of Th2/Th1 was 1.525. Results indicated that immunotherapy with modified LWDHW-1217A or 1217B can enhance the T_H_1 cell population and suppress the T_H_2 cell population. The ratio of Th2/Th1 was also noted to have decreased much less than treatment with saline. After immunotherapy, the cell viability was evaluated by flow cytometry with Propidium iodide (PI) staining which is a fluorescent dye binding to the nucleic acid of dead cells. The expressed fluorescence greater than 10^4^ in the PI staining is regarded as dead cells. The results showed that the percentage of cell viability was close to 95.0% and not significantly changed in the 1217A or 1217B-treated cells compared to the Naïve or Saline group (Fig. 8F), the results of three independent repetitions were presented in Fig. 8G. This suggest that the mixed compound of TCM-1217A or 1217B do not process a significant toxic effect on asthmatic mice and do not cause a significant increase in cell death.

### Effects of modified LWDHW-1217A and 1217B on pulmonary function of Airway Hyperresponsiveness (AHR)

**Fig. 9:**
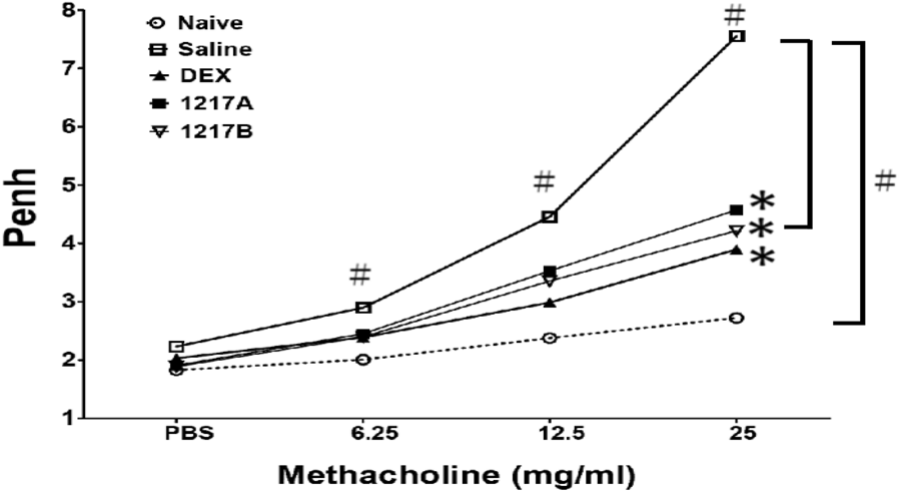
Differences in airway hyperresponsiveness (AHR) to methacholine were measured among these groups after immunotherapy. Pulmonary function of mice was measured after intratracheal challenge with allergen-nDer p 2 in the Naïve group and immunotherapy groups. Methacholine plays the role of a bronchoconstriction agent at a concentration of 6.25, 12.5, and 25 mg/ml. PBS Inhalation was the baseline of pulmonary function. The AHR to methacholine was measured 30 min after the intratracheal challenge, and the AHR presented as enhanced Pause (Penh) values with means ± SD. #: *p* < 0.05 compared to the Naïve group; *: *p* < 0.05 compared to the saline group

The pulmonary function of mice was evaluated by airway hypersensitivity to methacholine of whole-body plethysmography, which was measured one hour after nDer p 2 allergen IT challenges. The responses exposed to rising doses of methacholine (PBS, 6.25, 12.5, and 25.00 mg/ml) in five groups of mice were generated. Respiratory parameters such as enhanced pause (Penh), pause, peak inspiratory flow, peak expiratory flow, tidal volume, breathing frequency, end-inspiratory pause, and end-expiratory pause were recorded. Since airway constriction was mainly related to the change in Penh, the Penh value was used to evaluate the repression effects of 1217A and 1217B on pulmonary function. In the group of immunotherapy with saline after nDer p 2 sensitization, the Penh values were significantly increased after the methacholine challenge at the dose of 6.25, 12.5, and 25 mg/ml compared with those of naïve mice (Fig. 9; *p* < 0.05). When mice were immunotherapy with modified LWDHW-1217A or 1217B, the Penh significantly decreased after the methacholine challenge at the dose of 12.5, and 25 mg/ml compared to those with saline (Fig. 9; *p* < 0.05). There was also a significant reduction of Penh in airway hypersensitivity after methacholine challenge at the dose of 12.5 mg and 25 mg in the group treated with Dexamethasone compared to those with saline. Although the effect was also observed in the group of modified LWDHW-1217A, it was less than those treated with modified LWDHW-1217B. Overall, the Penh values were the lowest in the group when immunotherapy with Dexamethasone at the 25 mg/ml methacholine evocation. But there is no obvious difference between the group of 1217A, 1217B, and Dexamethasone. Results showed the Penh was significantly upregulated in the saline group at 25 mg/ml methacholine, but the Penh value was significantly decreased in the 1217B groups (Fig. 9; *p* < 0.05). Results indicated that immunotherapy with modified LWDHW-1217A or 1217B can alleviate the inflammatory response of pulmonary function triggered by mite allergen.

### Effects of modified LWDHW-1217A and 1217B on bronchus pathology

**Fig. 10:**
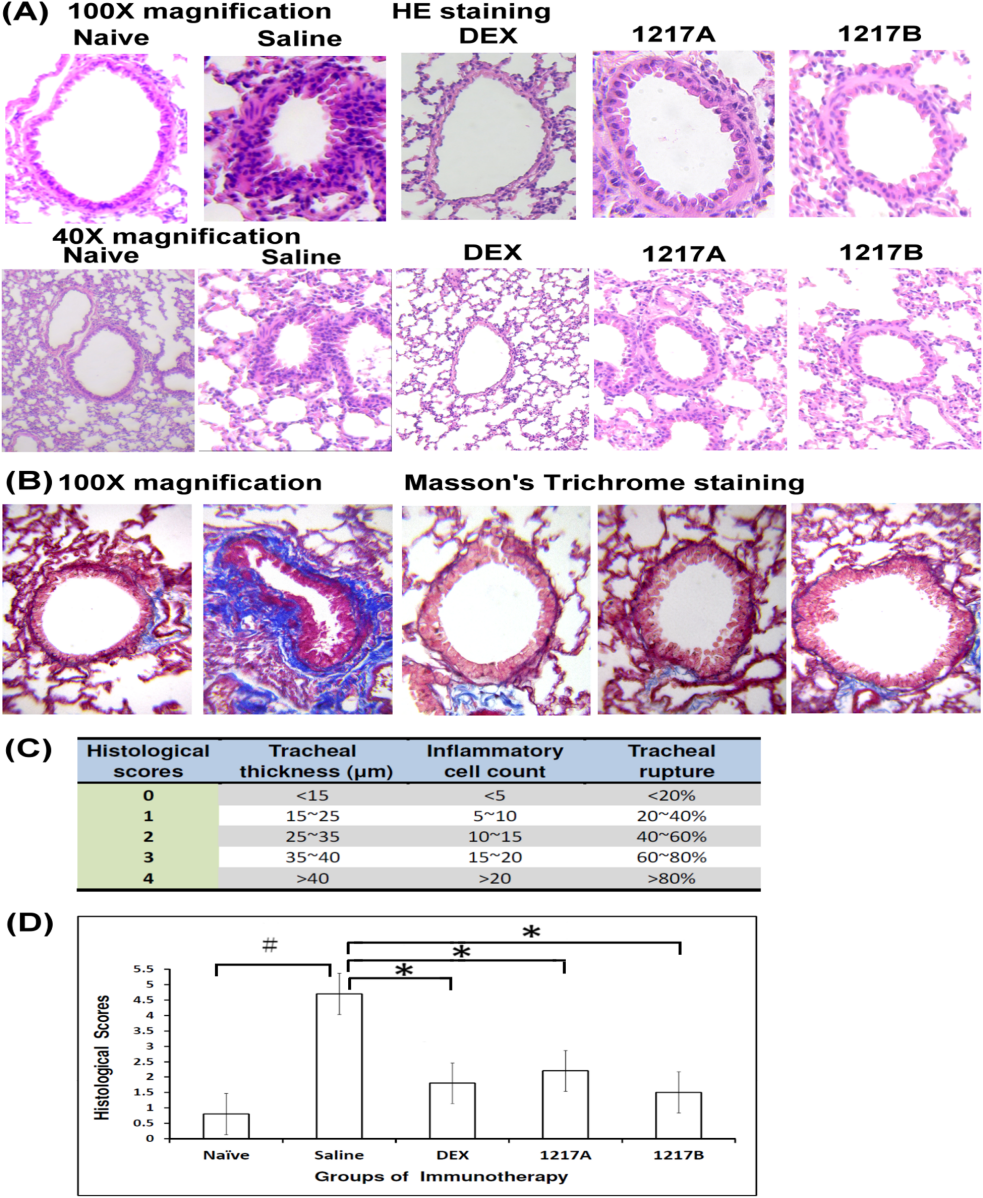
Pathology variety in mouse lungs from different groups acquired immunotherapy with TCM-1217A or 1217B after sensitization with mite allergen. **A** Pathology images of each group are presented respectively. Lung tissue cut at 5 μm thickness and stained with hematoxylin–eosin (100X and 40X magnification). Representative photomicrographs from mice treated with Naïve and immunotherapy with saline, DEX, 1217A, and 1217B. **B** 5-μm thick lung tissue sections were stained with Masson’s Trichrome of the collagen-rich fibrotic region present in blue for fibrosis detection (100X magnification). **C** The histologic score for each field is shown in terms of tracheal thickness, inflammatory cell count, and tracheal rupture. **D** Semiquantitative evaluation of lung pathology based on hematoxylin–eosin stain and evaluated with tracheal thickness, inflammatory cell count, and tracheal rupture. Ten visual fields of each mouse were observed, and the pathological features in the lung among different groups were presented as histological scores. #: *p* < 0.05 compared to the Naïve group; *: *p* < 0.05 compared to the saline group

The lung histopathology with hematoxylin–eosin staining and Masson’s Trichrome staining was performed to determine whether immunotherapy with modified LWDHW-1217A or 1217B affected bronchus pathology or airway fibrosis. The epithelium damage in the airway and inflammatory cell infiltration were compared among the naïve group and all experiment groups after immunotherapy with modified LWDHW-1217A, 1217B, or Dexamethasone. Representative photomicrographs among the naïve group and all experiment groups are presented respectively with 100X and 40X magnification in Fig. 10A. The intratracheal challenge with allergen Der p 2 was performed after the IP sensitization and treatment with saline, the results of pulmonary physiology showed that the bronchus pathology with the obvious infiltration of granulocytes (Fig. 10A). Both tracheal thickness and tracheal rupture were raised in the group treated with saline after allergen sensitization when compared to the naïve group (Fig. 10A). The Masson’s trichrome histochemical stain was used to determine the degree of collagen deposition around the airway section. We sought to investigate whether in vivo immunotherapy of modified LWDHW-1217A or 1217B would reduce the severity of lung fibrosis. The administration resulted in a significant reduction of collagen deposition around small bronchi and a notable decrease in the number of cells infiltrating the airways. The histological scores for each perspective were based on tracheal thickness (µm), inflammatory cell count (cell units), and tracheal rupture (%) (Fig. 10C). The lung histopathology was calculated by tracheal thickness, inflammatory cell count, and tracheal rupture; therefore, the histological scores were significantly higher in the saline group than in the naïve group (Fig. 10A; *p* < 0.05). Semiquantitative evaluation of lung pathology was based on hematoxylin–eosin stain and evaluated with tracheal thickness, inflammatory cell count, and tracheal rupture. There was a reduction of airway inflammatory cell infiltration and a decreased level of tracheal damage in the group of immunotherapies with modified LWDHW-1217A or 1217B when compared with those of saline (Fig. 10D; *p* < 0.05). There was also a reduction of airway inflammatory cell infiltration and epithelium damage in the tracheal after immunotherapy with Dexamethasone when compared with those of saline (Fig. 10D; *p* < 0.05). There was a trend of significant improvement in the bronchus pathology from mouse lungs in these immunotherapy groups, including modified LWDHW-1217A, 1217B, and Dexamethasone. Results indicated that immunotherapy with modified LWDHW-1217A or 1217B can abate the bronchus inflammation and attenuate airway fibrosis in the animal model of allergic asthma triggered by mite allergen.

## Discussion

Allergic diseases, including asthma, allergic rhinitis, and atopic dermatitis, occur worldwide and are more prevalent in westernized countries. Chronic immunological diseases are a growing public health concern. They are caused by unfavorable immune responses to allergens, resulting in sensitization, memory T and B cell development, IgE production, and inflammatory mediator release. Allergic symptoms occur when specific IgE on mast cells and basophils are cross-linked by allergens, leading to histamine and inflammatory mediator release [27]. Indoor allergens like mites can trigger allergic diseases through sensitization and provocation [28]. Inhaling mite allergens can alter airway epithelium and cause allergic symptoms, leading to airway allergy [29]. Prolonged exposure to dust mite allergens causes airway inflammation, inflammatory mediator release, and trachea remodeling, resulting in irreversible damage. Current medicine often cannot fully control mite-induced allergic symptoms.

More than 36 different allergic components that induce IgE in allergic patients and approximately 32 allergens in HDM have been identified based on IgE-binding frequency, protein sequence homology, and biochemical function [30]. The major IgE frequencies of mite allergens in allergic patients are different due to the discrepancies in countries, regions, human genomes, and survey seasons. Der p 2 is a major source of mite allergens and a leading cause of respiratory disorders such as allergic asthma, airway hypersensitivity, and impaired lung function [7]. Der p 2 is a major mite allergen processing non-proteolytic characteristics whose structure is very stable so that it does not easily decompose and exists in the surrounding environment for a long time even after mite death [6]. The Der p 2 exhibits molecular mimicry of structural homology and similar biological activity with Myeloid Differentiation-2 (MD-2), which is a lipopolysaccharide(LPS)-binding member of the Toll-like receptor-4 (TLR-4) signaling complex, resulting in Der p 2 can facilitate TLR-4 signaling through direct interactions to trigger innate immunity and its allergenicity [11]. The major mite allergen Der p 2 significantly elevated nerve growth factor (NGF) production in the BALF, activation of alveolar macrophages and mast cells, eosinophilic infiltration, and airway inflammation through the p38 mitogen-activated protein kinase (MAPK) pathway and increasing ROS production [31]. Der p 2 induced secretions of granulocyte–macrophage colony-stimulating factor (GM-CSF), IL-6, IL-8, monocyte-chemotactic protein (MCP)-1, and macrophage inflammatory protein-3alpha (MIP-3alpha), and intercellular adhesion molecule (ICAM)-1 in human bronchial epithelial cells resulting in it causes the inflammation in lung epithelium and aggravates respiratory airway diseases [32].

The stratagem of allergy prevention includes allergen avoidance, pharmacotherapy, and allergen-specific immunotherapy. Allergen-specific immunotherapy is an effective treatment used for allergic diseases which alleviates immoderate immune responses and relieves the pathogenesis progression. The traditional form of immunotherapy incorporates the administration of progressively raised quantities of relevant allergens until the dose approaches an effective degree for inducing immunologic tolerance. Crude allergen extracts used in immunotherapy present challenges due to the potential for unexpected events, such as anaphylactic reactions derived from the tricky standardization of allergen levels within extracts [33]. Allergen crude extracts can have inconsistent compositions due to variations in manufacturers, batches, and extraction methods. Mite crude extracts contain various components, including mite bodies, fecal pellets, mite allergens, eggs, surface microbes, and intracellular bacterial symbionts, which may act as immunogens. Recombinant allergens produced through gene recombination technology can minimize side effects caused by crude extracts and enhance therapeutic effectiveness. Two mutants of the clinically important dust mite allergen Der p 2 with consistent low IgE binding have good potential as prophylactic hypoallergenic vaccine candidates for immunotherapy in a murine model [34]. The Der p 2-based peptide vaccine shows reduced allergenicity of IgE-related and basophil activation when compared with the wild-type allergen and modified Der p 2 allergen may be used for prophylactic vaccination [35]. The local nasal immunotherapy with recombinant Der p 2 (rDer p 2) in conjunction with fungal immunomodulatory protein (FIP) can downregulate the airway inflammation in both *D. pteronyssinus-* and rDer p 2-sensitization mice. This combination also even can enhance the Th1-biased immunogenicity and reduceTh2-biased allergenicity, it suggests the major allergen Der p 2 and in conjunction with immune adjuvant FIP might be a good alternative therapy for allergy which cause by dust mite [36]. In this study, we demonstrated that the mite major allergen-Der p 2 induces airway inflammation in the asthmatic murine model and that hypersensitivity to methacholine can be modulated by TCM of modified LWDHW-1217A and 1217B. The immunoglobulin productions of Der p 2-specific IgE and IgG1 in sera and inflammatory cytokine productions of IL-5 and IL-13 in the airway BALF were significantly decreased, the results overall in this study indicated the modified LWDHW-1217A or 1217B has the potential to down-regulate the inflammatory tendency caused by the mite allergen.

For centuries, Chinese clinicians have used TCMs to treat allergic diseases, particularly bronchial asthma, with significant improvements in clinical outcomes. LWDHW administration was found to prevent airway bronchoconstriction, reduce eosinophil infiltrations in BALF, and lower production of inflammatory mediators (histamine and LTC4) in asthmatic guinea pigs [37]. Although LWDHW administration only partially improved clinical parameters such as symptom score, medication score, and peak expiratory flow rate in asthmatic children, this study found that it could increase total T-cells and decrease B-cells [37]. Previous study did not observe changes in the lymphocyte differentiation subsets of T-cells and specificity immunoglobulin productions of B-cells. However, this study analyzed the expression of the T_H_1-type cytokine (IFN-γ) and T_H_2-type cytokine (IL-4) produced in CD4 + cells and found that modified LWDHW-1217A or 1217B immunotherapy can enhance the T_H_1 cell population and suppress the T_H_2 cell population. Traditional LWDHW treatment significantly inhibited both RNA and protein levels of T_H_2-type cytokines (IL-4, IL-5, IL-10, and IL-13) and T_H_1-type cytokines (IL-2 and IFN-γ) in human peripheral blood lymphocytes in vitro [26]. Investigations into T_H_1 and T_H_2 responses in asthmatic mice focused on cytokine productions in sera and BALF, lung tissue gene expression, phosphorylation of STAT6, STAT1, and ERK, activation of GATA3, and percentages of IFN-γ^+^/CD4^+^ and IL-4^+^/CD4^+^ in T cells (Figs. 4, 6, 7, and 8) in this study. Overall, modified LWDHW-1217A or 1217B enhanced T_H_1 cell-related activities while suppressing T_H_2 cell-related activities, possibly due to the prolonged oral administration in *vivo* allowing for better immune adjustment compared to one-day treatment with traditional LWDHW in *vitro*. The second reason is that the modified LWDHW-1217A or 1217B contains three Chinese traditional medicines (*Radix Dioscorea*, *Alismatis Rhizoma*, and *Scutellaria baicalensis*) with anti-inflammatory effects, as indicated in Table 1. The combination of these Chinese traditional medicines is based on their potential to inhibit inflammatory responses and recommendations from Chinese medical physicians.

TCMs, including Anti-asthma Herbal Medicine Intervention (ASHMI), Modified Mai-Men-Dong-Tang (mMMDT), Ding Chuan Tang (DCT), and STA-1(combination of mMMDT and LWDHW), have favorable therapeutic effects with fewer side effects, making them valuable and easily accessible resources from conventional medicine for treating asthma [38–41]. Increasing evidence shows that TCM formulas have substantial pharmacological activities, abundant ingredients, and structural diversity suitable for treating asthma in a complex and multi-target manner. Persistent mild-to-moderate asthmatic patients who received mMMDT therapy experienced noticeable improvements in lung function (FEV1), serum total IgE, dust mite specific IgE, and symptom scores after 4 months of treatment [39]. The herbal formula mMMDT may improve lung function, ameliorate clinical symptoms, and has no reported drug-related adverse effects [39]. Despite limited understanding of the detailed mechanisms and active ingredients of mMMDT, the evidence supports their efficacy, safety, low cost, and favorable compliance characteristics. As a result, mMMDT may serve as an effective alternative to Western medicine for mild-to-moderate asthma patients, in addition to existing therapies [39]. The STA-1 formula (combination of mMMDT and LWDHW) could effectively suppresses clinical symptoms, improves pulmonary function (FEV1), and inhibits the synthesis of total and mite-specific IgE in mild-to-moderate asthmatic patients, with no reported adverse side effects during the intervention period [40]. In this clinical trial, the detailed mechanism of pharmacological actions and curative ingredients of the STA-1 formula were unclear [40]. In summary, TCM therapy for asthmatic patients offers several advantages, including clinical effectiveness, affordability, and low risk of life-threatening or severe side effects. These TCM formulas provide improved efficacy and safety, particularly in special populations like children with mild-to-moderate asthma.

In this study, formula 1217A and 1217B were used, which differ only in the content of *Alismatis Rhizoma* (3 g in 1217A and 9 g in 1217B). Both formulas contain four herbs, including *Poria cocos*, *Radix Dioscorea*, and *Scutellaria baicalensis* (Table 1). The primary difference between formulas 1217A and 1217B is the amount of *Alismatis Rhizoma*. TCM *Poria cocos* ethanol extract has been shown to decrease the production of inflammatory mediators such as nitric oxide, prostaglandin E2 (PGE2), interleukin-1β, and tumor necrosis factor-alpha (TNF)-α by suppressing the NF-kappaB signaling pathway in macrophages stimulated with lipopolysaccharide [42]. Both formulas of 1217A and 1217B had increased the content of *Poria cocos* in this study, its purpose is to strengthen the pharmacological actions of anti-inflammatory effects after exposure to mite allergens. The Yam (*Dioscorea*) were selected to add in the formula of 1217A or1217B due to it has been confirmed anti-inflammatory effects and to modulate inflammatory mediators by inhibiting the NF-κB pathway. Inhibition of NF-κB subsequently down-regulates inflammatory markers such as COX-2, iNOS, TNF-α, and IL-1β, therefore these effects may be attributed to the antioxidant and anti-inflammatory nature of Yam (*Dioscorea*) [22]. The pretreatment with *Alismatis* ethanol extracts can effectively prevent the development of neutrophil lung infiltration in an LPS-induced lung inflammation of mouse models with acute lung injury [23]. The *Alismatis Rhizoma* extract is involved in the inhibition of pro-inflammatory transcription factor NF-kB and activation of anti-inflammatory transcription factor Nrf2 so that it can attenuate lung inflammation [23]. The *Alismatis Rhizoma* extract represents anti-inflammatory effects that can be developed as a potential therapeutic for acute or chronic lung inflammations caused by pathogens or allergens. The active compound wogonin from *Scutellaria baicalensis* (Skullcap) can suppress IL-4 production ex vivo and down-regulate IgE and IL-5 production on OVA-induced Th2 immune responses in vivo, it suggests the wogonin from *Scutellaria baicalensis* possesses anti-inflammatory properties so that can be applied as a therapeutic agent for IgE- or IL-5 mediated allergic disorders [24].

The formula 1217A and 1217B used in this study notably included two effective ingredients, *Poria cocos,* and *Scutellaria baicalensis,* the composition of herbal complexes Saiboku-to (TJ-96) [43]. TJ-96, an herbal medicine tested on asthmatic patients in a double-blind and randomized clinical trial, has shown anti-inflammatory effects on bronchial eosinophilic infiltration. Results suggest that TJ-96 may reduce eosinophilic inflammation and offer therapeutic potential for asthma treatment [43]. Saiboku-to contains three triterpenoids (magnolol, dihydroxy-dihydromagnolol, and liquiritigenin) that have been shown to significantly inhibit leukotriene C4, making them a potential treatment option for bronchial asthma [44]. Saiboku-to's three active components can inhibit asthmatic effects through anti-inflammatory responses, indicating clinical efficacy and potential as an anti-allergic agent [44]. Baicalein, extracted from Scutellaria root, has been shown to suppress human eotaxin mRNA expression in fibroblasts, which is associated with eosinophil recruitment, demonstrating its pharmacological efficacy [45]. The previous study demonstrated the extracts of yam *Dioscorea* could downregulate ovalbumin-induced allergic response in mice and reduce inflammatory parameters including IgE, intestinal edema, and mucus production, suggesting the yam *Dioscorea* has the potential to prevent and treat allergies associated with food allergens [46]. The methanol extracts from *Alismatis Rhizoma* not only inhibit antibody-mediated allergic reactions but also influence immune cell reactions, it exhibits the inhibitory effect on contact dermatitis in mice which serves as a useful agent for the treatment of allergic reactions [47]. The yam *Dioscorea* and *Alismatis Rhizoma* had been added to the formula 1217A and 1217B in this study, and the productions of mite allergen-specific immunoglobulins (IgE and IgG1), Th2-cytokines (IL-5 and IL-13), and expressions of inflammatory genes (*IL-4*, *IL-5*, and *IL-13*), neutrophil chemotactic chemokine (*IL-8*), and T_H_2-related transcription factor (*GATA-3*) can be down-regulated after immunotherapy with 1217A or 1217B. The study found that immunotherapy with modified LWDHW-1217A or 1217B can increase TH1 cell population, decrease TH2 cell population, and reduce the ratio of Th2/Th1 compared to the saline group. Immunotherapy with 1217A or 1217B also significantly improved pulmonary function and reduced bronchial inflammation in lung histopathology, as evidenced by decreased collagen deposition and cell infiltration around small bronchi. The study showed that immunotherapy with modified LWDHW-1217A or 1217B can reduce bronchial inflammation and airway fibrosis in an animal model of allergic asthma triggered by mite allergen. The study also evaluated the difference between the effects of 1217A or 1217B in the asthmatic model, and although 1217B appeared to have better therapeutic efficacy, there was no statistical difference between the two. However, more research is needed to confirm the detailed mechanism of pharmacological actions and the curative ingredients of TCM formula 1217A or 1217B.

Administering methacholine to the subcarinal airways via the bronchial artery caused dose-dependent bronchoconstriction and tracheal segment contraction. This method is commonly used to diagnose bronchial airway hyperresponsiveness, asthma severity, airway inflammation, and tracheal obstruction in animals with asthma. Methacholine inhalation increases bronchial sensitivity to inflammatory mediators and leads to contraction, ultimately reducing FEV1 and increasing Penh [48]. Bronchial asthma is characterized by airway inflammation and hypersensitivity. The Methacholine provocation test is highly sensitive for diagnosing asthma severity in allergic patients with bronchial hyperresponsiveness. However, other pulmonary diseases may also cause bronchial hyperresponsiveness, such as COPD, cystic fibrosis, bronchitis, allergic rhinitis, or congestive heart failure. To achieve a more comprehensive understanding, additional physiological variations should be explored in animal models of asthma, such as immunoglobulin generation, cytokine production, Th1/Th2 polarization, gene expression in lung tissue, and bronchial pathology. Immunotherapy with modified LWDHW-1217A or 1217B reduced airway inflammation and airway hyperresponsiveness to methacholine inhalation. This suggests that these formulas could be used as therapeutic agents for treating allergic bronchial asthma. Although Th1 or Th2 polarization in CD4 + cells were observed in peripheral blood, further investigation of IFN-γ or IL-4 expression in lung and mediastinal lymph nodes is needed to better understand the immunomodulatory effects of TCM formula 1217A or 1217B.

In allergic reactions, allergens activate T cells to differentiate into Th2 cells and secrete IL-4, IL-5, and IL-13, leading to allergen-specific IgE production. Treatment with 1217A or 1217B decreased Der p 2-specific IgE and IL-5 levels in BAL fluid and sera, downregulated the Th2/Th1 ratio, and reduced total cell numbers, macrophages, lymphocytes, eosinophils, and neutrophils in BAL fluid. This inhibition of Th2 cytokines resulted in decreased IgE binding to FcεRI on basophil or mast cells, minimizing the allergic reaction and further preventing inflammatory cell infiltration. Therefore, 1217A and 1217B could be useful for treating allergic asthma.

Comprehensively observing the overall results, the therapeutic efficacy of TCM formula 1217A or 1217B appears to be similar to the dexamethasone in the asthmatic animal model. Dexamethasone is a commonly prescribed corticosteroid medication for the treatment of asthma due to its anti-inflammatory effects. However, long-term use of dexamethasone can have adverse effects, including osteoporosis, weight gain, increased blood sugar, and weakened immune system. In contrast, the modified LWDHW of 1217A or 1217B has a relatively improved safety profile, making it a potentially safer alternative for long-term use. The TCM formula 1217A or 1217B takes a more holistic approach to healthcare, which includes a focus on lifestyle changes and dietary modifications in addition to herbal remedies. It incorporates several natural herbs that have been used in Chinese medicine for centuries and can potentially provide additional health benefits. The TCM formula 1217A or 1217B has the potential for personalized treatment which it can be tailored to each patient's specific needs and symptoms, making it a potentially more effective treatment option for individual patients. The modified LWDHW of 1217A or 1217B can provide an alternative treatment option for asthma patients who cannot tolerate corticosteroids or prefer to use natural remedies.

## Conclusion

Traditional Chinese Medicine seems to be better therapeutic effects and has been used to treat acute and chronic asthma. Few studies mentioned the therapeutic effects of Traditional Chinese Medicine of LWDHW on the mite allergen-induced asthmatic animal model. The detailed mechanisms of modified LWDHW of 1217A or 1217B on improvements in immunomodulation and physiological variety of asthmatic animal models remain unclear. Even there are fewer evidence-based studies demonstrating mechanisms by which the LWDHW could be useful for asthmatic patients. This study aimed to investigate the modified LWDHW of 1217A or 1217B whether with therapeutic potential for treating allergic asthma in mite allergen Der p 2-sensitized asthmatic mice. At least ten active ingredients were contained in the formula of modified LWDHW- 1217A and 1217B including 1-allantoin, 2-loganin, 3-catalpol, 4-baicalin, 5- alisol acetate B, 6-methyl gallate, 7-baicalein, 8-wogonin, 9-saponin, and 10-pachymic acid which order by the retention time. Results indicated that the immunoglobulin generations (Der p 2 specific- IgE and IgG1), inflammatory cytokine productions (IL-5 and IL-13) in the Sera and BALF could be down-regulated, and the Th1-cytokine productions (IL-12 and IFN-γ) be increased after immunotherapy with modified LWDHW of 1217A or 1217B. The inflammatory cell infiltrations (macrophages, eosinophils, and neutrophils) in the airway and the expressions of T_H_2-related genes (*IL-4*, *IL-5*, and *IL-13*), T_H_2-related transcription factor (*GATA-3*), and neutrophil chemotactic chemokine (*IL-8*) in the lung tissue of asthmatic mice were significantly decreased after the immunotherapy. The Th1/Th2 polarization had been identified that the IL-4^+^/CD4^+^ T cells were downregulated and IFN-γ^+^/CD4^+^ T cells were increased. The pulmonary function of airway hyperresponsiveness to methacholine inhalation was significantly decreased in Penh values of the 1217A or 1217B groups. There were significant improvements in the bronchus histopathology after immunotherapy with 1217A or 1217B which were evaluated by tracheal thickness, inflammatory cell count, and tracheal rupture of mouse lung. This study revealed the modified LWDHW of 1217A or 1217B could regulate the immune responses and improve pulmonary function. It suggests that 1217A or 1217B have the potential for use as a therapeutic modality for the treatment of mite allergen Der p 2-induced allergic asthma.

## Data Availability

The data used to support the findings of this study are available from the corresponding author upon reasonable request.

## Abbreviations

modified LWDHW: Modified Liu-Wei-Di-Huang-Wan
TCM: Traditional Chinese Medicine
nDer p 2: Natural Der p 2
MD-2: Differentiation factor-2
LPS: Lipopolysaccharide
TLR: Toll-like receptor
PBMC: Peripheral blood mononuclear cells
DEX: Dexamethasone
IP: Intraperitoneal
IT: Intratracheal
AHR: Airway hyperresponsiveness
BALF: Bronchoalveolar lavage fluid
FITC: Fluorescein isothiocyanate
PMA: Phorbol myristate acetate
SDS-PAGE: Sulfate–polyacrylamide gel electrophoresis
PVDF: Polyvinylidene difluoride
PBST: PBS containing 1% Tween-20
Nrf2: Erythroid 2 related factor 2
COX-2: Cyclooxygenase-2
